# Micellar catalysis-enabled sustainable ppm Au-catalyzed reactions in water at room temperature†
†Electronic supplementary information (ESI) available. See DOI: 10.1039/c7sc02405c



**DOI:** 10.1039/c7sc02405c

**Published:** 2017-07-20

**Authors:** Piyatida Klumphu, Camille Desfeux, Yitao Zhang, Sachin Handa, Fabrice Gallou, Bruce H. Lipshutz

**Affiliations:** a Department of Chemistry and Biochemistry , University of California , Santa Barbara , California 93106 , USA . Email: lipshutz@chem.ucsb.edu; b Chimie Paris Tech , Paris , France; c Department of Chemistry , University of Louisville , Kentucky 40292 , USA . Email: sachin.handa@louisville.edu; d Novartis Pharma , Basel , Switzerland

## Abstract

Several ppm level gold-catalyzed reactions enabled by the ligand HandaPhos can be performed at room temperature in aqueous nanoreactors composed of the surfactant Nok. Variously substituted allenes undergo cycloisomerization leading to heterocyclic products in good yields. Likewise, cyclodehydration is also illustrated under similar conditions, as is an intermolecular variant, hydration of terminal alkynes. Recycling of the catalyst and reaction medium is also illustrated. A low *E* factor associated with limited solvent use and therefore, waste generation, documents the greenness of this process.

## Abstract 2

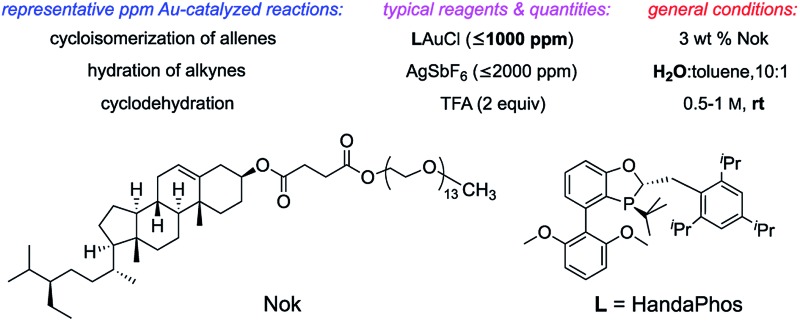
Several ppm level gold-catalyzed reactions enabled by the ligand HandaPhos can be performed at room temperature in aqueous nanoreactors composed of the surfactant Nok.

## Introduction

Notwithstanding the enormous progress made in gold-catalyzed reactions over the past decade, and the associated extensive and comprehensive reviews that appear on almost a yearly basis,^1^ there are two aspects associated with this chemistry that seem unaddressed: (1) existence of a general technology that offers opportunities to use this precious metal at levels below the traditional 1–5 mol% range, and (2) reaction conditions that are environmentally responsible, where use of organic solvents is essentially eliminated, especially chlorinated solvents, and catalysis can be performed at ambient temperatures rather than requiring an investment of energy usually in the form of heat. Relatively few reports on the development of ppm level gold catalysis have appeared, and these tend to focus on one specific type of reaction.^2^ In 2009, Nolan was the first to describe hydrations of alkynes that took place with ≤1000 ppm of NHC-complexed AuCl together with AgSbF_6_, and while a breakthrough documenting the potential, the educts were relatively simple and the conditions rather vigorous (*vide infra*).^3^ A few years ago, Zhang and co-workers reported use of ppm levels of gold that led to additions of acids to alkynes forming enol esters enabled by the clever design of a new ligand.^4^ Here again, an organic medium (Ph-F) at close to reflux temperatures over time, and without recycling, are characteristic features of this process. Thus, in addition to a few other isolated cases,^1^ these document the absence of a reported study describing ppm level gold catalysis that appears to be amenable to several types of reactions under green chemistry conditions.

**Fig. 1:**
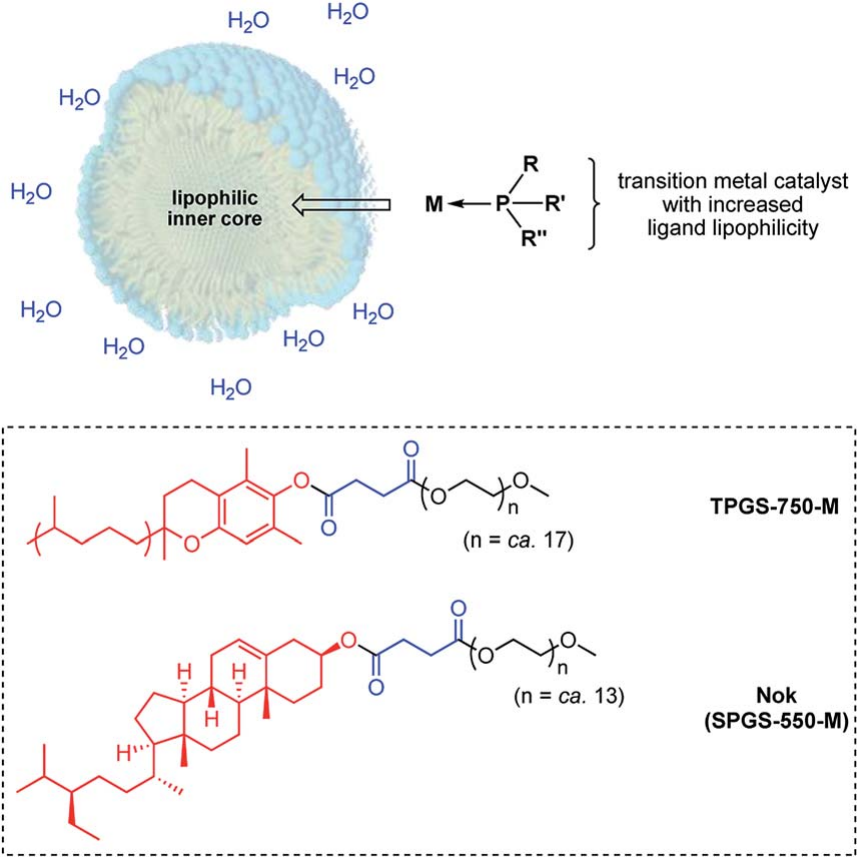
Formation of a HandaPhos-Au catalyst for use at the ppm level under micellar catalysis conditions.

One approach to lowering the required levels of precious metals involved in catalytic processes is to take advantage of the higher concentrations of both water-insoluble reactants and catalysts preferentially found within the inner cores of nanomicelles in water (Fig. 1).^5^ The extent to which the occupants reside within these nanoreactors, as opposed to their dynamic exchange between nanomicelles, depends upon their binding constants. The greater the binding constant for a given ligated gold catalyst, the more time spent within each micelle and hence, the less needed for catalysis. This requires that in addition to consideration of the common elements fundamental to ligand design, such as steric, stereoelectronic, and conformational effects, as well as donicity, lipophilicity may be an important consideration,^6^ which is otherwise meaningless for catalysis run in organic solvents.^1*b*^

**Scheme 1:**
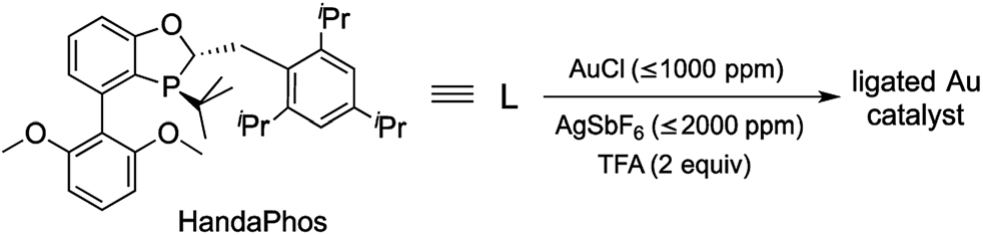
Formation of a HandaPhos-Au catalyst for use at the ppm level under micellar catalysis conditions.

These considerations have led to the design of the recently introduced ligand (racemic) HandaPhos^6^ (Scheme 1), shown to function in the form of a 1 : 1 complex with palladium as a means of effecting Suzuki–Miyaura cross-couplings at the ppm level of metal in water at room temperature. To determine if HandaPhos technology applies to other precious metal chemistry, we have turned to cationic gold in an effort to provide not only ppm level catalysis of several typical Au-catalyzed reactions, but to make such a process relatively environmentally benign. It was anticipated that both the electron-rich and bulky nature of this ligand would contribute to its effectiveness in gold catalysis, especially for intramolecular cyclizations where protodeauration is known to be rate-determining.^7^ In this report, we disclose technology that, indeed, provides a new Au(i) catalyst that can be used, and recycled, at the ≤1000 ppm (0.1 mol%)^8^ level under aqueous micellar conditions.

## Results and discussion

Given the rich history of gold activation of allenic arrays,^9^ especially involving cycloisomerizations that have been advanced from early studies by Hashmi,^10^ Krause,^11^ and then Widenhoefer^12^ and Toste,^13^ and others, we began our investigation by optimizing several reaction parameters associated with cyclizations of allenic alcohols using the designer surfactant Nok^14^ in water at rt. Unlike prior art that relies oftentimes on 5 mol% of a Au catalyst in chlorinated media, we set as the upper limit 1000 ppm (*i.e.*, 0.1 mol%) Au in water at rt. As shown in Table 1, cyclization of an allenol to dihydropyran required the presence of both TFA as well as a silver salt. While initial results were poor using either AgOTf or AgBF_4_, moderate results were observed by switching to AgSbF_6_ in a 1 : 1 ratio with ligated (tetrahydrothiophene)AuCl. The yield could be increased dramatically to 98% by increasing the amount of this same silver salt to 2000 ppm in the presence of the acid activator TFA (2 equiv.).^15^ Control experiments documented the crucial role that each ingredient plays in this chemistry; neither ligated gold nor silver salt, nor TFA alone led to any reaction (NR). Likewise, use of AgSbF_6_ (2000 ppm) + TFA gave NR, with or without the presence of HandaPhos. However, upon addition of (tetrahydrothiophene)AuCl, full activity was restored, suggesting dual catalysis by this ppm Au/Ag combination.^16^

**Table 1:** Optimization of catalyst conditions for cyclization reaction in water at room temperature

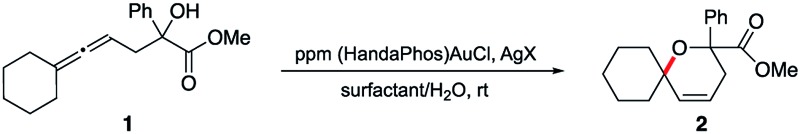		
Entry	Conditions	Result
1	LAuCl (1000 ppm) + AgBF_4_ (1000 ppm)	NR
2	LAuCl (1000 ppm) + AgOTf (1000 ppm)	NR
3	LAuCl (1000 ppm) + AgSbF_6_ (1000 ppm)	NR
4	LAuCl (1000 ppm) + AgBF_4_ (1000 ppm) + TFA (1 equiv.)	56% (5 d)
5	LAuCl (1000 ppm) + AgOTf (1000 ppm) + TFA (1 equiv.)	Trace
6	LAuCl (1000 ppm) + AgSbF_6_ (1000 ppm) + TFA (1 equiv.)	71% (4 d)
**7**	**LAuCl (1000 ppm) + AgSbF_6_ (2000 ppm) + TFA (2 equiv.)**	**98%**

Additional screening as to the choice of surfactant included the background reaction “on water” (Table 2). Clearly, cyclization could be achieved under such micelle-free conditions; however, the extent of conversion was low.

**Table 2:** Screening reaction media

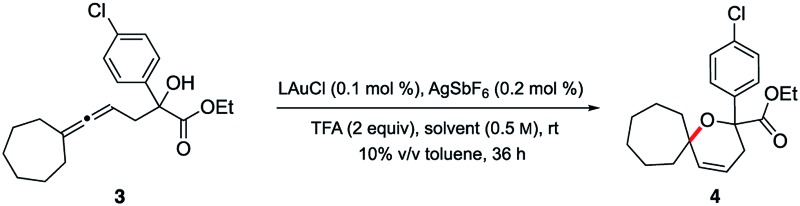		
Entry	Solvent	Yield (%)
1	Water	54
**2**	**3 wt% Nok**	**90**
3	3 wt% TPGS-750-M	86
4	DCM	Incomplete (after 4 d)
5	Toluene	Incomplete (after 4 d)

Switching from Nok to the alternative, vitamin E-based derivative, TPGS-750-M,^17^ afforded roughly comparable results. Poor conversions were noted in both organic solvents DCM and toluene even after four days.

**Scheme 2:**
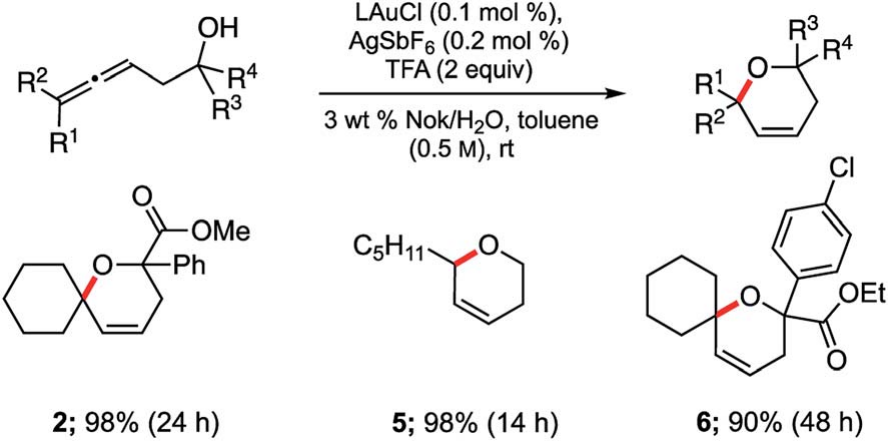
Representative examples of cyclizations of allenic alcohols catalyzed by ppm Au ligated by HandaPhos (L).

Application of these newly established conditions to three additional allenic alcohols led to the corresponding dihydropyrans as shown in Scheme 2. In all cases, cyclization took place smoothly and gave the expected products in high isolated yields. Both di- and tri-substituted allenes are amenable to this Au-catalyzed process in water. Use of small percentages of co-solvent (toluene, 10% v/v)^18^ was found to have a beneficial effect on the extent of conversion and hence, yield.

**Scheme 3:**
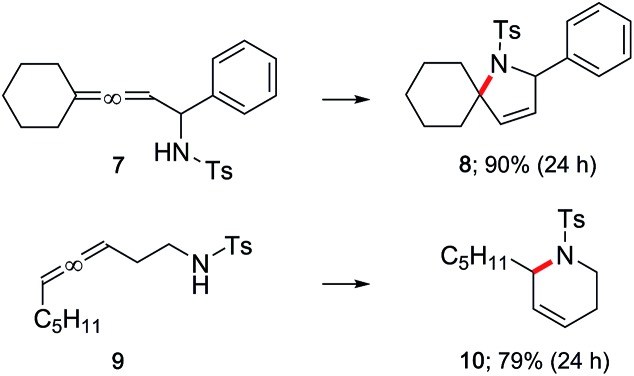
Representative examples of cyclizations of allenic sulfonamides catalyzed by ppm Au ligated by HandaPhos (L) [conditions: see Scheme 2].

These optimized conditions are also applicable to aminoallenes, as illustrated in Scheme 3. No conversion was observed with free amines, where the high affinity of an amino group for gold can inhibit the reaction as can its potential protonation by TFA. Derivatization as the sulfonamide (with TsCl) was sufficient to overcome this undesirable association, leading to smooth cyclization. Both α- and β-aminoallenes **7** and **9** were reactive and the corresponding cyclized 5- and 6-membered rings **8** and **10** were obtained in good-to-excellent yields. Moreover, mono- and di-substituted aminoallenes readily participated.

**Scheme 4:**
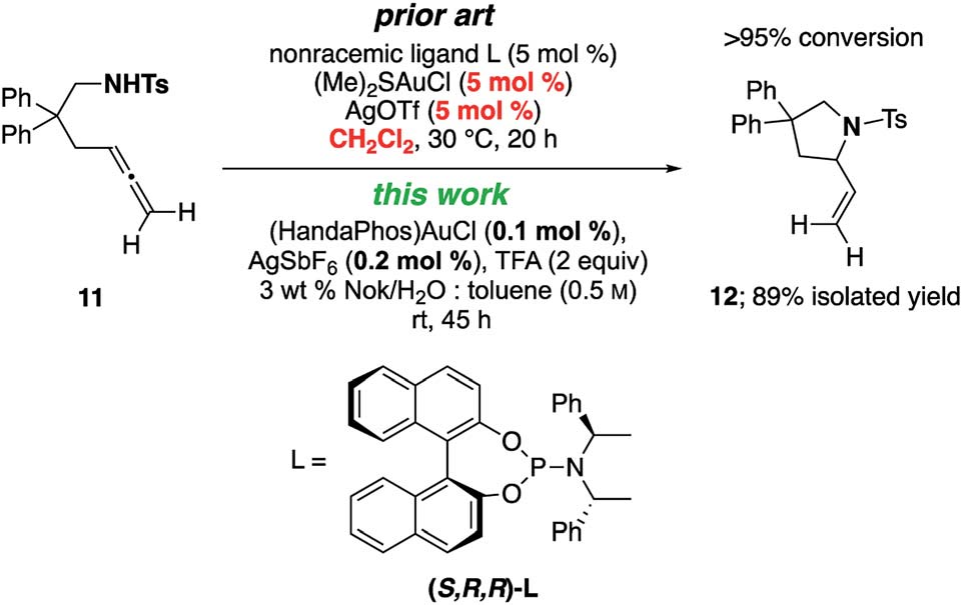
Literature comparison: cycloisomerization of a γ-aminoallene.

A γ-aminoallene was also cyclized, this example serving as a direct comparison with known literature conditions.^19^ By contrast, cyclization under micellar conditions reflects a significant drop in the amount of gold catalyst and associated silver salt, as well as avoidance of a chlorinated reaction solvent (Scheme 4).

**Fig. 2:**
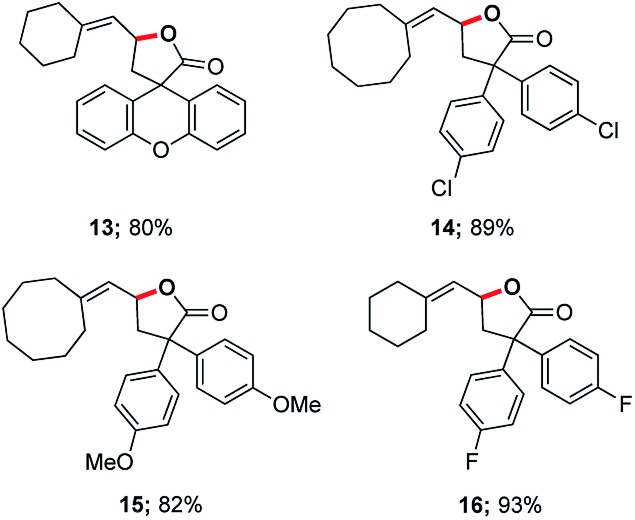
Representative examples of cyclizations of allenic acids catalyzed by ppm Au ligated by HandaPhos.

In 2014, asymmetric gold-catalyzed lactonization was reported by us wherein 3 mol% of a gold complex was employed, also enabled by micellar catalysis in water at room temperature.^20^ Cyclizations of the same type of allenic acids were re-examined using ppm levels of a (racemic) gold catalyst (Fig. 2). Although longer reaction times were required, the expected products (**13–16**) were obtained in comparable yields.

**Scheme 5:**
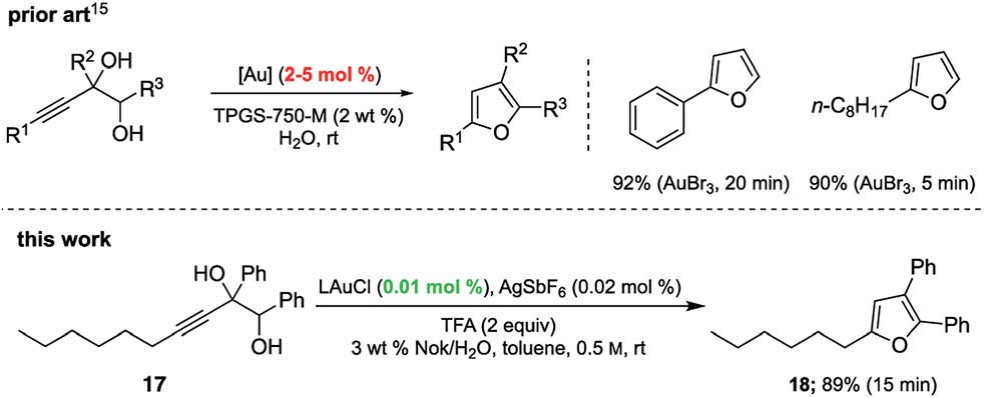
Comparison of cyclodehydration reactions between prior art and HandaPhos (L) technology.

Gold-catalyzed cyclodehydrations of variously function-alized hydroxy- and amino-allenes in water were pioneered by the Krause group, first reported back in 2009, which utilized chloroauric acid (HAuCl_4_) as catalyst.^11*d*^ Limitations due to substrate insolubility in water led to their switch to amphiphiles under aqueous micellar conditions.^21^ The catalyst of choice was AuBr_3_ (2–5 mol%), used in the presence of 2 M NaCl. Advantages noted included a significant reaction rate acceleration, as well as minimization of organic waste *via* elimination of organic solvents. The same type of ring formation leading to substituted furans could be accomplished using HandaPhos technology where 200–500 times less gold need be used (*i.e.*, 100 ppm, before recycling) to realize the same outcome, in 15 minutes at rt (Scheme 5).

**Fig. 3:**
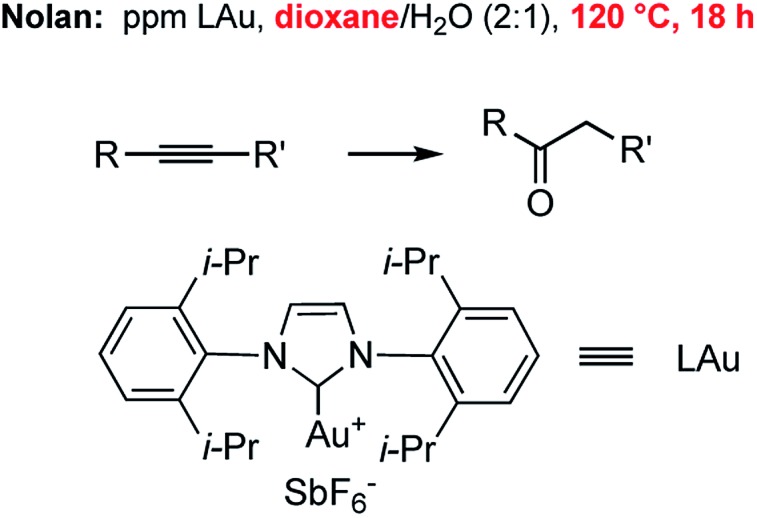
Nolan's approach to ppm level Au-catalyzed hydration of alkynes.

**Scheme 6:**
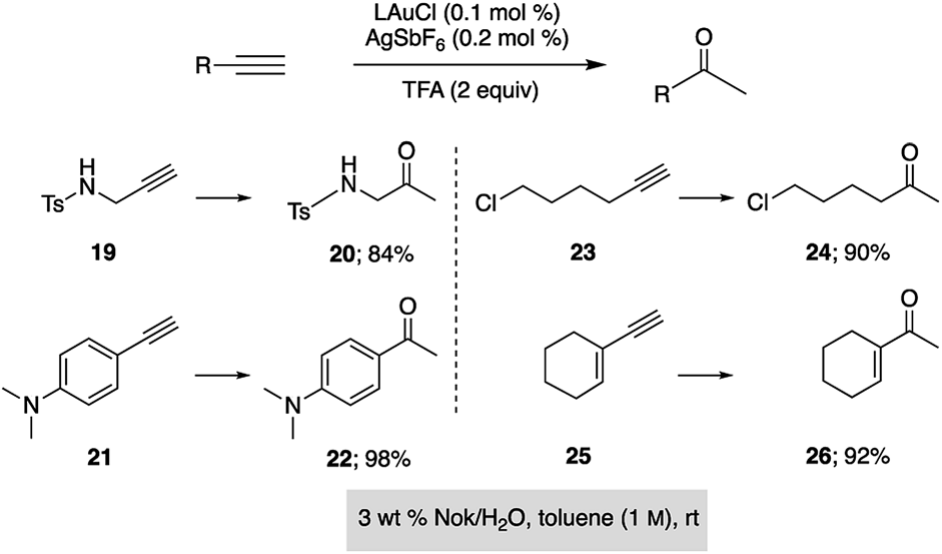
Application of HandaPhos technology to ppm level Au-catalyzed hydration of terminal alkynes.

Hydration of alkynes represents a fundamental route to methyl ketones.^22^ Nolan's approach,^3^ as illustrated in Fig. 3, employed low levels of an NHC Au complex, with added AgSbF_6_, typically between 100–1000 ppm, although these were performed in refluxing aqueous dioxane over an 18 hour time frame. Alternatively, use of our standard conditions on terminal alkynes led to functionalized methyl ketones in aqueous nanomicelles at rt over 24 h in high yields (Scheme 6).

**Scheme 7:**
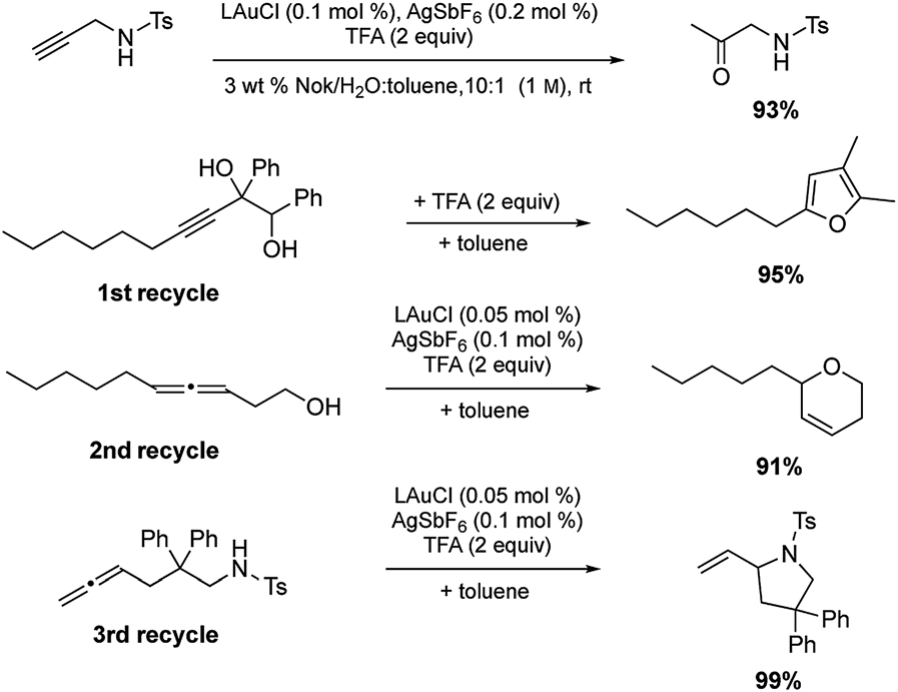
Recycling of the aqueous reaction mixture [L = HandaPhos].

Among the virtues of this technology is the opportunity to recycle the entire reaction mixture following an “in flask” extraction of the product using a minimum of a single (recyclable) organic solvent. Moreover, the same reaction need not be used in each recycling step. As shown in Scheme 7, initial hydration of a sulfonamide could be followed by a cyclodehydration, followed by two successive, albeit distinct, cyclizations. After the first two reactions, additional catalyst (500 ppm Au and 1000 ppm Ag) was required, presumably due to deactivation from earlier processing. Nonetheless, the total investment of gold for these four reactions was 0.2 mol%.

**Scheme 8:**
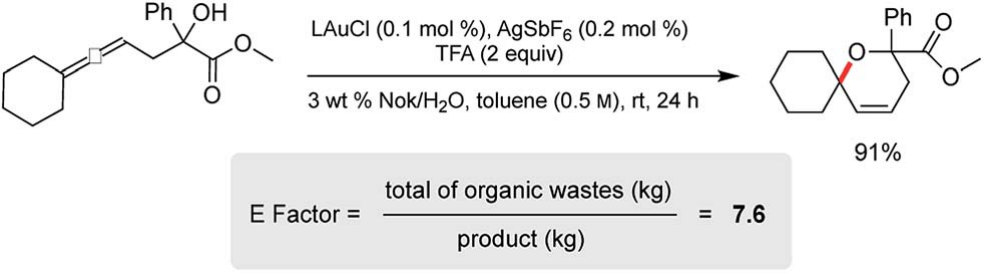
*E* factor determination.

Cyclization of an allenic alcohol as a representative substrate (Scheme 8) led to a calculated *E* factor of 7.6 on the basis of organic solvent used (see ESI†). This signifies a considerable improvement over values (25–100) typically associated with the pharmaceutical industry^23^ and is in line with numbers seen previously for related reactions in micellar media.^24^

## Conclusions

In summary, several representative examples of gold-catalyzed reactions of both an intra- and intermolecular nature have been shown to be amenable to aqueous micellar catalysis that enables use of ppm levels of catalyst, rather than the typical investment of 1–5 mol%. These new procedures minimize exposure to organic solvents, eliminate energy input in the form of heat, and facilitate recycling of the entire reaction mixture. Moreover, associated low *E* factors indicate minimal amounts of hazardous waste generation. In the composite, these data suggest that gold catalysis appears to be well suited to become yet another tool in the arsenal of green synthetic chemistry.
