# Supplementary material for: Micellar catalysis-enabled sustainable ppm Au-catalyzed reactions in water at room temperature
Source: Chem Sci. 2017 Jul 20;8(9):6354–8. doi: 10.1039/c7sc02405c (PMC5628599; doi:10.1039/c7sc02405c)
Supplement: Supplementary file 1 [file SC-008-C7SC02405C-s001.pdf]

Electronic Supplementary Material (ESI) for Chemical Science.
This journal is © The Royal Society of Chemistry 2017

Micellar catalysis applied to sustainable *ppm* Au-catalyzed reactions in water at room temperature

Piyatida Klumphu,[†] Camille Desfeux,[§] Sachin Handa,[‡] Yitao Zhang, Fabrice Gallou,[#] and Bruce H. Lipshutz*[†]

† Department of Chemistry and Biochemistry, University of California, Santa Barbara, California 93106, United States

§ Chimie Paris Tech, Paris, France

‡ Department of Chemistry, University of Louisville, Kentucky, 40292, United States

[#] Novartis Pharma, Basel, Switzerland

Supporting information

Table of Contents

1. General information S2
2. Experimental procedures S2
3. Spectral data S12
4. References S35

1. General Information

All reactions were carried out in a sample vial (4 mL) equipped with a Teflon-coated magnetic stir bar. HPLC grade water was degassed by sparging with argon prior to use. Solutions of 3.0 wt % Nok and other surfactants in degassed water were made and stored in a glass container with a septum on the bench-top. HandaPhos ligand was synthesized according to the original procedures.[1] Gold pre-catalyst was prepared by procedures below and stored in a dry box prior to use. Starting materials were prepared applying reference procedures. Column chromatography was carried out using Silica gel 60 (230 – 400 mesh). TLC analysis was done using TLC Silica gel 60 F_{254} glass plates. ^{1}H and ^{13}C NMR spectra were obtained in $CDCl_3$ using 400 MHz, 500 MHz or 600 MHz NMR spectrometer. High-resolution mass spectral analyses were obtained using a double-focusing magnetic sector instrument for EI and a quadrupole/TOF instrument (API) for ESI.

2. Experimental Procedures

Preparation of gold pre-catalyst

(HandaPhos)Au(I) chloride. (Tetrahydrothiophene)gold(I) chloride was generated from tetrahydrothiophene and auric acid according to a known procedure.[2] A 10 mL round-bottom flask equipped with a Teflon-coated magnetic stir bar and septum was charged with HandaPhos (27.3 mg, 0.05 mmol) and (tetrahydrothiophene)gold(I) chloride (16.0 mg, 0.05 mmol). A rubber septum was added to the flask, which was degassed and filled with argon. The flask was covered with aluminum foil to protect it from light. Anhydrous dichloromethane (2 mL) was added via syringe and the reaction was stirred for 2 h. After, the solvent was removed under vacuum, the product was put under high vacuum overnight to remove trace amounts of solvent and tetrahydrothiophene. A white solid was obtained (35.0 mg, 90%). ^{1}H NMR (400 MHz, $CDCl_3$) δ 7.53 (dd, J = 11.8, 4.3 Hz, 1H), 7.38 (t, J = 8.4 Hz, 1H), 7.01 (s, 2H), 6.91 (ddd, J = 7.4, 4.8, 2.6 Hz, 2H), 6.72 (d, J = 8.4 Hz, 1H), 6.58 (d, J = 8.4 Hz, 1H), 4.95 (dd, J = 10.3, 3.7 Hz, 1H), 3.88 (s, 3H), 3.70 (d, J = 7.6 Hz, 3H), 3.30 – 3.20 (m, 2H), 2.92 (ddt, J = 37.3, 13.8, 6.8 Hz, 3H), 1.53 (s, 2H), 1.25 (dd, J = 9.0, 3.5 Hz, 12H), 1.18 (d, J = 6.8 Hz, 6H), 0.96 (d, J = 17.2 Hz, 9H); ^{13}C NMR (101 MHz, $CDCl_3$) δ 163.23, 157.36, 157.08, 147.55, 147.12, 139.96, 134.26, 130.49, 128.32, 128.23, 125.65, 125.57, 121.24, 113.10, 111.68, 104.79, 103.21, 77.19, 55.84, 55.26, 34.72, 34.43, 34.09, 34.07, 33.94, 29.56, 25.95, 25.88, 24.77, 24.02, 23.93; ^{31}P NMR (162 MHz, $CDCl_3$) δ 59.97.

Gold pre-catalyst. HandaPhos-gold(I) chloride (0.8 mg, 0.001 mmol) and silver(I) hexafluoroantimonate (0.7 mg, 0.002 mmol) were charged under an argon atmosphere into a 5 mL microwave vial containing a Teflon-coated magnetic stir bar and a rubber septum. The vial was covered with aluminum foil to protect the compounds from light. Anhydrous dichloromethane (1 mL) was added via syringe and the reaction was stirred for 15-20 min prior to use.

General Procedures for ppm Au-catalyzed cycloisomerization of allenes

To a 4 mL glass vial equipped with Teflon-coated magnetic stir bar, 100 µL of gold pre-catalyst in DCM solution (1000 ppm or 0.1 mol %) was added and the DCM was removed *in vacuo*. Allene (0.1 mmol) was added to the vial followed by toluene (200 µL), 3 wt % Nok/H_2O (0.2 mL, 0.5 M), and trifluoroacetic acid (23 mg, 0.2 mmol). The resulting mixture was stirred at rt until completion. Thin-layer chromatography was used to follow the reaction. After completion, the product was exacted with EtOAc (3 x 0.1 mL) and the solvent was evaporated under vacuum. Crude product was purified via column chromatography using hexane/EtOAc to afford pure material.

Hydroxyallene (Figure 3)

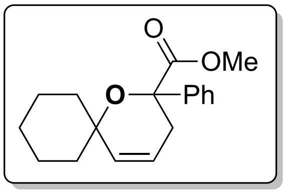

Methyl 2-phenyl-1-oxaspiro[5.5]undec-4-ene-2-carboxylate (2)

Following the general procedure using methyl 5-cyclohexylidene-2-hydroxy-2-phenylpent-4-enoate (27.3 mg, 0.1 mmol), the reaction was allowed to stir for 24 h. After column chromatography, the product was obtained as a yellow liquid (26.8 mg, 98%). IR ($CDCl_3$) 3031, 2931, 2856, 1732, 1448, 1262, 1192, 1056, 824, 719 cm^{-1}; ^1H NMR (600 MHz, $CDCl_3$) δ 7.55 – 7.51 (m, 2H), 7.33 (t, J = 7.7 Hz, 2H), 7.28 – 7.23 (m, 1H), 5.89 (ddd, J = 10.1, 6.4, 2.2 Hz, 1H), 5.76 (dd, J = 10.3, 2.3 Hz, 1H), 3.60 (s, 3H), 3.09 (dd, J = 16.5, 6.4 Hz, 1H), 2.18 (dt, J = 16.5, 2.5 Hz, 1H), δ 1.96 – 1.83 (m, 2H), 1.73 – 1.27 (m, 8H); ^{13}C NMR (126 MHz, $CDCl_3$) δ 174.39, 143.39, 133.77, 128.25, 127.44, 124.59, 121.72, 78.07, 74.86, 51.95, 39.27, 36.23, 33.12, 25.49, 22.21, 21.58; HRMS (EI) calcd for $C_{18}H_{22}O_3$ $[M]^+$ 286.1569, found 286.1571 (Δ = 0.2 mDa, 0.7 ppm).

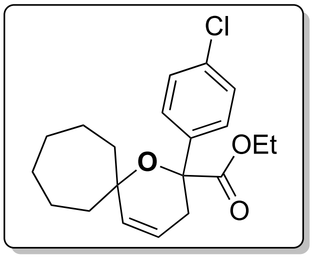

Ethyl 2-(4-chlorophenyl)-1-oxaspiro[5.6]dodec-4-ene-2-carboxylate (4, Table 2)

Following the general procedure using ethyl 2-(4-chlorophenyl)-5-cycloheptylidene-2-hydroxypent-4-enoate (35.9 mg, 0.1 mmol), the reaction was allowed to stir for 48 h. After column chromatography, the product was obtained as a yellow liquid (33.0 mg, 92%). IR ($CDCl_3$) 3040, 2925, 2856, 1728, 1490, 1260, 1091, 1046, 830, 724 cm^{-1}; ^1H NMR (600 MHz, $CDCl_3$) δ 7.46 (d, *J* = 8.6 Hz, 2H), 7.28 (d, *J* = 8.6 Hz, 2H), 5.85 – 5.77 (m, 2H), 4.11 – 4.01 (m, 2H), 3.03 (dd, *J* = 16.3, 6.1 Hz, 1H), 2.14 – 2.06 (m, 1H), 1.98 – 1.37 (m, 12H), 1.15 (t, *J* = 7.1 Hz, 3H). ^{13}C NMR (126 MHz, $CDCl_3$) δ 173.59, 141.97, 135.29, 133.21, 128.30, 126.28, 120.26, 78.55, 78.25, 61.20, 42.82, 39.59, 32.83, 29.23, 29.19, 22.32, 22.10, 13.86; HRMS (EI) calcd for $C_{20}H_{25}O_3Cl$ $[M]^+$ 348.1492, found 348.1490 (Δ = -0.2 mDa, -0.6 ppm).

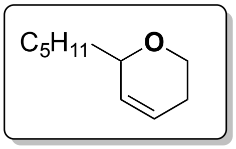

6-*n*-Pentyl-3,6-dihydro-2*H*-pyran (5)

Following the general procedure using deca-3,4-dien-1-ol (18.3 mg, 0.1 mmol), the reaction was allowed to stir for 14 h. After column chromatography, the product was obtained as a colorless liquid (17.9 mg, 98%). ^1H NMR (600 MHz, $CDCl_3$) δ 5.83 – 5.77 (m, 1H), 5.61 (dd, *J* = 10.3, 1.5 Hz, 1H), 4.03 (d, *J* = 1.9 Hz, 1H), 3.95 (ddd, *J* = 11.0, 5.6, 2.0 Hz, 1H), 3.67 – 3.59 (m, 1H), 2.30 – 2.20 (m, 1H), 1.94 – 1.84 (m, 1H), 1.56 – 1.21 (m, 8H), 0.87 (t, *J* = 6.9 Hz, 3H).[3]

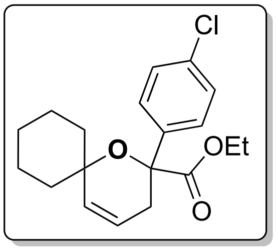

Ethyl 2-(4-chlorophenyl)-1-oxaspiro[5.5]undec-4-ene-2-carboxylate (6)

Following the general procedure using ethyl 2-(4-chlorophenyl)-5-cyclohexylidene-2-hydroxypent-4-enoate (33.3 mg, 0.1 mmol), the reaction was allowed to stir for 48 h. After column chromatography, the product was obtained as a yellow liquid (30.0 mg, 90%). IR ($CDCl_3$) 3035, 2932, 2857, 1728, 1490, 1259, 1203, 1091, 1058, 1009, 756, 711 cm^{-1} ^1H NMR (600 MHz, $CDCl_3$) δ 7.47 (d, *J* = 8.6 Hz, 2H), 7.29 (d, *J* = 8.6 Hz, 2H), 5.87 (ddd, *J* = 10.0, 6.5, 2.2 Hz, 1H), 5.74 (dd, *J* = 10.3, 2.3 Hz, 1H), 4.08 – 4.00 (m, 2H), 3.07 (dd, *J* = 16.4, 6.5 Hz, 1H), 2.12 (ddd, *J* = 15.5, 9.0, 6.5 Hz, 1H), 1.94 – 1.81 (m, 2H), 1.76 (d, *J* = 13.4 Hz, 1H), 1.62 (dd, *J* = 18.7, 9.1 Hz, 2H), 1.56 – 1.41 (m, 3H), 1.38 – 1.27 (m, 2H), 1.13 (t, *J* = 7.1 Hz, 3H); ^{13}C NMR (126 MHz, $CDCl_3$) δ 173.59, 141.97, 135.29, 133.21, 128.30, 126.28, 120.26, 78.55,

78.25, 61.20, 42.82, 39.59, 32.83, 29.23, 29.19, 22.32, 22.10, 13.86; HRMS (EI) calcd for $C_{19}H_{23}O_3Cl$ $[M]^+$ 334.1336, found 334.1344 (Δ = 0.8 mDa, 2.4 ppm).

Aminoallene (Figure 4)

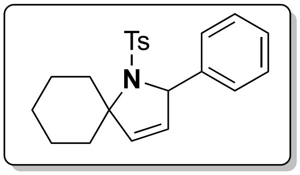

2-Phenyl-1-tosyl-1-azaspiro[4.5]dec-3-ene (8)

Following the general procedure using *N*-(3-cyclohexylidene-1-phenylallyl)-4-methylbenzene-sulfonamide (37.2 mg, 0.1 mmol), the reaction was allowed to stir for 24 h. After column chromatography, the product was obtained as a white solid (33.4 mg, 90%). mp 150-152 °C; IR ($CDCl_3$) 3073, 3035, 2925, 2865, 1598, 1494, 1456, 1330, 1154, 1092, 676, 585, 547 cm^{-1} ^1H NMR (600 MHz, $cdcl_3$) δ 7.38 (d, *J* = 8.2 Hz, 2H), 7.16 – 7.10 (m, 5H), 6.99 (d, *J* = 8.0 Hz, 2H), 6.22 (dd, *J* = 6.4, 2.0 Hz, 1H), 5.53 (dd, *J* = 6.4, 1.9 Hz, 1H), 5.50 (t, *J* = 1.9 Hz, 1H), 2.82 – 2.73 (m, 1H), 2.55 (td, *J* = 13.2, 4.3 Hz, 1H), 2.29 (s, 3H), 2.01 – 1.96 (m, 1H), 1.86 – 1.67 (m, 4H), 1.59 – 1.30 (m, 3H).; ^{13}C NMR (126 MHz, $CDCl_3$) δ 142.11, 140.05, 139.31, 131.22, 128.76, 128.11, 127.97, 127.75, 127.42, 77.13, 71.01, 38.72, 37.23, 25.27, 24.79, 21.32; HRMS (EI) calcd for $C_{22}H_{25}NO_2S$ $[M]^+$ 367.1606, found 367.1609 (Δ = 0.3 mDa, 0.8 ppm).

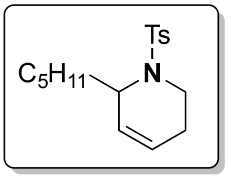

6-*n*-Pentyl-1-tosyl-1,2,3,6-tetrahydropyridine (10)

Following the general procedure using *N*-(deca-3,4-dien-1-yl)-4-methylbenzenesulfonamide (31.7 mg, 0.1 mmol), the reaction was allowed to stir for 24 h. After column chromatography, the product was obtained as a colorless liquid (25.0 mg, 79%). IR ($CDCl_3$) 3035, 2926, 2857, 1598, 1456, 1343, 1329, 1157, 1094, 710, 686, 553 cm^{-1}; ^1H NMR (600 MHz, $cdcl_3$) δ 7.68 (d, *J* = 8.2 Hz, 2H), 7.21 (d, *J* = 8.0 Hz, 2H), 5.57 (dt, *J* = 10.4, 7.9 Hz, 2H), 4.24 (s, 1H), 3.81 (dd, *J* = 14.5, 6.0 Hz, 1H), 3.15 – 3.06 (m, 1H), 2.38 (s, 3H), 1.80 – 1.17 (m, 10H), 0.86 (t, *J* = 6.9 Hz, 3H); ^{13}C NMR (126 MHz, $CDCl_3$) δ 142.86, 138.72, 129.42, 128.19, 126.98, 124.63, 53.70, 38.30, 35.08, 31.69, 25.74, 22.99, 22.52, 21.47, 14.02; HRMS (EI) calcd for $C_{17}H_{25}NO_2S$ $[M]^+$ 307.1606, found 307.1611 (Δ = 0.5 mDa, 1.6 ppm)

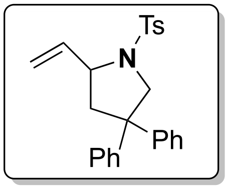

4,4-Diphenyl-1-tosyl-2-vinylpyrrolidine (12, Scheme 1)

Following the general procedure using *N*-(2,2-diphenylhexa-4,5-dien-1-yl)-4-methylbenzene-sulfonamide (25.7 mg, 0.1 mmol), the reaction was allowed to stir for 45 h. After column chromatography, the product was obtained as a yellow liquid (22.8 mg, 89%). ^{1}H NMR (400 MHz, cdcl$_3$) δ 7.62 (t, *J* = 10.9 Hz, 2H), 7.34 – 7.10 (m, 12H), 5.66 – 5.50 (m, 1H), 5.13 (t, *J* = 16.1 Hz, 1H), 5.00 (t, *J* = 11.2 Hz, 1H), 4.18 (d, *J* = 10.3 Hz, 1H), 4.10 (dd, *J* = 15.9, 8.9 Hz, 2H), 2.78 (dt, *J* = 15.8, 8.0 Hz, 1H), 2.51 – 2.37 (m, 4H).[4]

Allenic acid (Figure 5)

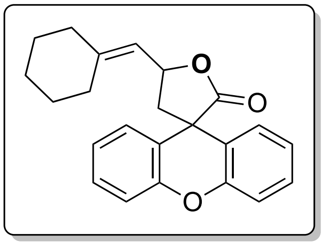

5-(Cyclohexylidenemethyl)-4,5-dihydro-2*H*-spiro[furan-3,9'-xanthen]-2-one (13)

Following the general procedure using 9-(3-cyclohexylideneallyl)-9,10-dihydroanthracene-9-carboxylic acid (34.8 mg, 0.1 mmol), the reaction was allowed to stir for 48 h. After column chromatography, the product was obtained as a viscous oil (27.8 mg, 80%). ^{1}H NMR (400 MHz, CDCl$_3$) δ 7.39 – 7.10 (m, 8H), 5.45 (ddd, *J* = 9.9, 8.7, 6.3 Hz, 1H), 5.25 (d, *J* = 8.5 Hz, 1H), 2.72 (dd, *J* = 13.4, 6.2 Hz, 1H), 2.34 – 2.08 (m, 4H), 1.72 – 1.44 (m, 6H).[5]

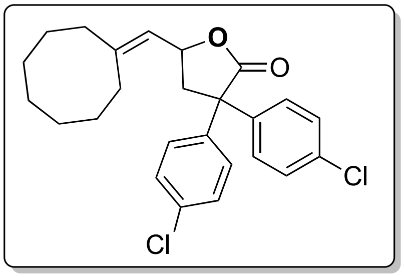

3,3-Bis(4-chlorophenyl)-5-(cyclooctylidenemethyl)dihydrofuran-2(3*H*)-one (14)

Following the general procedure using 2,2-bis(4-chlorophenyl)-5-cyclooctylidenepent-4-enoic acid (43.3 mg, 0.1 mmol), the reaction was allowed to stir for 48 h. After column chromatography, the product was obtained as a viscous oil (38.6 mg, 89%). ^{1}H NMR (400 MHz, $CDCl_3$) δ 7.41 – 7.19 (m, 8H), 5.27 (d, *J* = 8.9 Hz, 1H), 5.12 – 5.03 (m, 1H), 3.01 (dd, *J* = 13.2, 4.9 Hz, 1H), 2.64 (dd, *J* = 13.1, 10.5 Hz, 1H), 2.29 – 2.15 (m, 4H), 1.76 – 1.39 (m, 10H).[5]

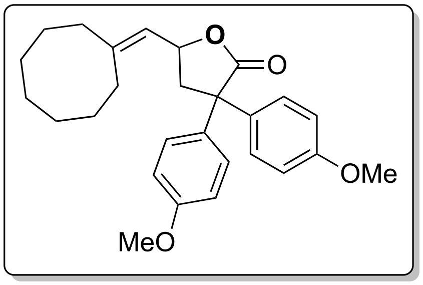

5-(Cyclooctylidenemethyl)-3,3-bis(4-methoxyphenyl)dihydrofuran-2(3*H*)-one (15)

Following the general procedure using 5-cyclooctylidene-2,2-bis(4-methoxyphenyl)pent-4-enoic acid (42.1 mg, 0.1 mmol), the reaction was allowed to stir for 48 h. After column chromatography, the product was obtained as a viscous oil (35.3 mg, 82%). ^{1}H NMR (400 MHz, $CDCl_3$) δ 7.33 (d, *J* = 8.8 Hz, 2H), 7.19 (d, *J* = 8.8 Hz, 2H), 6.91 (d, *J* = 8.8 Hz, 2H), 6.83 (d, *J* = 8.8 Hz, 2H), 5.32 – 5.27 (m, 1H), 5.12 – 5.03 (m, 1H), 3.80 (d, *J* = 18.1 Hz, 6H), 3.01 (dd, *J* = 13.1, 4.9 Hz, 1H), 2.62 (dd, *J* = 13.0, 10.5 Hz, 1H), 2.21 (d, *J* = 4.9 Hz, 4H), 1.57 (dd, *J* = 80.2, 11.7 Hz, 10H).[5]

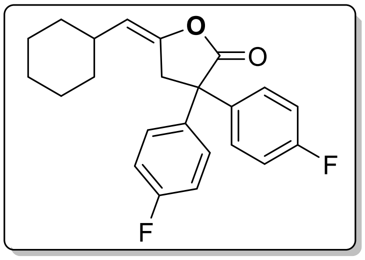

5-(Cyclohexylmethylene)-3,3-bis(4-fluorophenyl)dihydrofuran-2(3H)-one (16)

Following the general procedure using 5-cyclohexylidene-2,2-bis(4-fluorophenyl)pent-4-enoic acid (36.8 mg, 0.1 mmol), the reaction was allowed to stir for 48 h. After column chromatography, the product was obtained as a viscous oil (34.2 mg, 93%). ^{1}H NMR (400 MHz, $CDCl_3$) δ 7.39 – 7.32 (m, 2H), 7.30 – 7.22 (m, 2H), 7.11 – 7.04 (m, 2H), 7.03 – 6.95 (m, 2H), 5.26 (d, *J* = 8.5 Hz, 1H), 5.04 (ddd, *J* = 10.4, 8.6, 4.9 Hz, 1H), 3.01 (dd, *J* = 13.1, 4.9 Hz, 1H), 2.64 (dd, *J* = 13.1, 10.5 Hz, H), 2.38 – 2.18 (m, 4H), 1.72 – 1.41 (m, 6H).[5]

Dehydrative cyclization (Scheme 2)

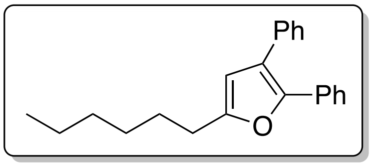

5-Hexyl-2,3-diphenylfuran (18)

To a 4 mL glass vial equipped with Teflon-coated magnetic stir bar, 9 µL of gold precatalyst in dichloromethane solution (100 ppm or 0.01 mol %) was added and the DCM was removed in vacuo. 1,2-Diphenyldec-3-yne-1,2-diol (28.0 mg, 0.089 mmol) was added to the vial followed by toluene (20 µL), 3 wt % Nok/H_2O (0.2 mL, 0.5M), and trifluoroacetic acid (21 mg, 0.17 mmol). The resulting mixture was stirred at rt for 10 min. After completion, the product was exacted with EtOAc (4 x 0.8 mL) and the solvent was evaporated under vacuum. Crude product was purified via column chromatography using hexane/EtOAc system afforded as a yellow liquid (25 mg, 94%). IR ($CDCl_3$) 3065, 3040, 2926, 2856, 1674, 1598, 1448, 1215, 1175, 932, 761, 693 cm^{-1}; ^1H NMR (500 MHz, $CDCl_3$) δ 7.54 (d, J = 7.4 Hz, 2H), 7.44 (d, J = 7.1 Hz, 2H), 7.36 (t, J = 7.4 Hz, 2H), 7.33 – 7.25 (m, 3H), 7.22 (t, J = 7.3 Hz, 1H), 6.19 (s, 1H), 2.72 (t, J = 7.6 Hz, 2H), 1.80 – 1.70 (m, 2H), 1.50 – 1.32 (m, 6H), 0.94 (t, J = 6.9 Hz, 3H).; ^{13}C NMR (126 MHz, $CDCl_3$) δ 198.68, 155.80, 153.88, 146.55, 136.06, 134.84, 134.41, 133.25, 131.59, 130.54, 129.12, 128.68, 128.61, 128.53, 128.29, 127.16, 126.97, 126.90, 125.93, 123.91, 122.95, 109.27, 43.52, 31.62, 28.97, 28.81, 28.08, 27.99, 23.79, 22.60, 22.46, 14.10; HRMS (EI) calcd for $C_{22}H_{24}O$ $[M]^+$ 304.1827, found 304.1821 (Δ = -0.6 mDa, -2.0 ppm).

General procedures of hydration of alkynes (Figure 7)

To a 4 mL glass vial equipped with Teflon-coated magnetic stir bar, 200 µL of gold pre-catalyst in dichloromethane solution (1000 ppm or 0.1 mol %) was added and the DCM was removed *in vacuo*. Alkyne (0.2 mmol) was added to the vial followed by toluene (200 µL), 3 wt % Nok/H_2O (0.2 mL, 1.0 M), and trifluoroacetic acid (46 mg, 0.4 mmol). The resulting mixture was stirred at rt for 24 h. After completion, the product was exacted with EtOAc (3 x 0.1 mL) and the solvent was evaporated off under vacuum. Crude product was purified via column chromatography using hexane/EtOAc.

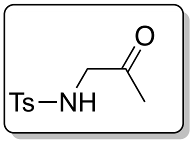

4-Methyl-*N*-(2-oxopropyl)benzenesulfonamide (20)

Following the general procedure using 4-methyl-*N*-(prop-2-yn-1-yl)benzenesulfonamide (42.4 mg, 0.2 mmol), the reaction was allowed to stir for 19 h. After column chromatography, the product was obtained as an off-white solid (38.8 mg, 84%). mp 91-95 °C;[6] ^{1}H NMR (500 MHz, $CDCl_3$) δ 7.74 – 7.70 (d, J = 8.0 Hz, 2H), 7.29 (d, J = 8.0 Hz, 2H), 5.35 (s, 1H), 3.84 (d, J = 4.5 Hz, 2H), 2.41 (s, 3H), 2.10 (s, 3H).[6]

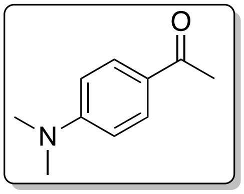

1-(4-(Dimethylamino)phenyl)ethan-1-one (22)

Following the general procedure using 4-ethynyl-*N*,*N*-dimethylaniline (29.2 mg, 0.2 mmol), the reaction was allowed to stir for 22 h. After column chromatography, the product was obtained as a red solid (32.3 mg, 98%). mp 87-89 °C;[7] ^{1}H NMR (600 MHz, $CDCl_3$) δ 7.84 (d, J = 9.0 Hz, 2H), 6.62 (d, J = 9.0 Hz, 2H), 3.03 (s, 6H), 2.48 (s, 3H).[8]

Recycle study (Figure 8)

1st reaction

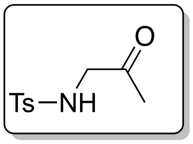

Followed the general procedure of hydration using 4-methyl-*N*-(prop-2-yn-1-yl)benzenesulfonamide (105.4 mg, 0.5 mmol), 500 μL of gold pre-catalyst in dichloromethane solution (1000 ppm or 0.1 mol %), toluene (500 μL), 3 wt % Nok/H_2O (0.5 mL, 1.0 M), and trifluoroacetic acid (114 mg, 1.0 mmol). The resulting mixture was stirred at rt for 24 h. After completion, the product was exacted with EtOAc (3 x 0.1 mL) and the solvent was evaporated under vacuum. Crude product was purified via column chromatography using hexane/EtOAc and pure product was obtained (106.9 mg, 93%). The aqueous phase containing the remaining

surfactant and catalyst is reused by addition of new substrate, toluene, and TFA to start the next cycle.

1st recycle

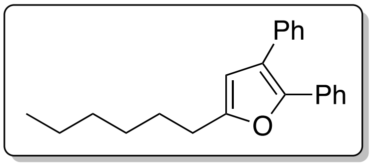

To a remaining aqueous phase containing catalyst and surfactant, 1,2-diphenyldec-3-yne-1,2-diol (160.0 mg, 0.5 mmol) was added into the reaction vial followed by addition of toluene (500 µL), and trifluoroacetic acid (114 mg, 1.0 mmol). The reaction was stirred for 15 mins after TLC confirmed completion. Product was obtained by in-flask extraction with EtOAc and the solvent was evaporated under vacuum. Crude product was purified via column chromatography using hexane/EtOAc and pure product was obtained (139.0 mg, 92%).

2nd recycle

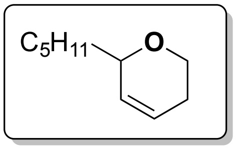

To a remaining aqueous phase, deca-3,4-dien-1-ol (75.5 mg, 0.5 mmol) was added into the reaction vial followed by addition of toluene (500 µL), and trifluoroacetic acid (114 mg, 1.0 mmol). Additional catalyst of 500 ppm (500 µL, 500 ppm) was added to catalyze the third reaction. The reaction was stirred for 24 h after TLC confirmed completion. Product was obtained by in-flask extraction with EtOAc and the solvent was evaporated under vacuum. Crude product was purified via column chromatography using hexane/EtOAc and pure product was obtained (68.5 mg, 91%).

3rd recycle

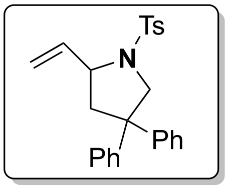

To a remaining aqueous phase, *N*-(2,2-diphenylhexa-4,5-dien-1-yl)-4-methylbenzene-sulfonamide (184.6 mg, 0.5 mmol) was added into the reaction vial followed by addition of toluene (500 µL), and trifluoroacetic acid (114 mg, 1.0 mmol). Additional catalyst (500 µL, 500

ppm) was added to catalyze the forth reaction. The reaction was stirred for 24 h after TLC confirmed completion. Product was obtained by in-flask extraction with EtOAc and the solvent was evaporated under vacuum. Crude product was purified via column chromatography using hexane/EtOAc and pure product was obtained (182.8 mg, 99%).

Calculation of E Factor

Following the general procedure of cycloisomerization of allenes using methyl 5-cyclohexylidene-2-hydroxy-2-phenylpent-4-enoate (28 mg, 0.1 mmol), the reaction was allowed to stir for 24 h. After completion, the product was obtained by extraction with EtOAc (200 μL, 180.4 mg) and crude product was purified by column chromatography. After column chromatography, the product was obtained as a colorless liquid (26 mg, 91%).

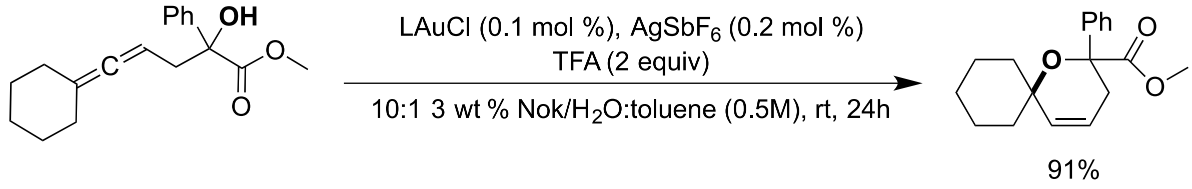

$$\text{E Factor} = \frac{\text{total of organic wastes (kg)}}{\text{product (kg)}}$$

$$= \frac{\text{toluene (co-solvent) + EtOAc (extraction)}}{\text{isolated pure product}}$$

$$= \frac{17.3 \text{ mg} + 180.4 \text{ mg}}{26 \text{ mg}}$$

$$= \mathbf{7.6}$$

3. Spectra

^{1}H NMR of (HandaPhos)AuCl

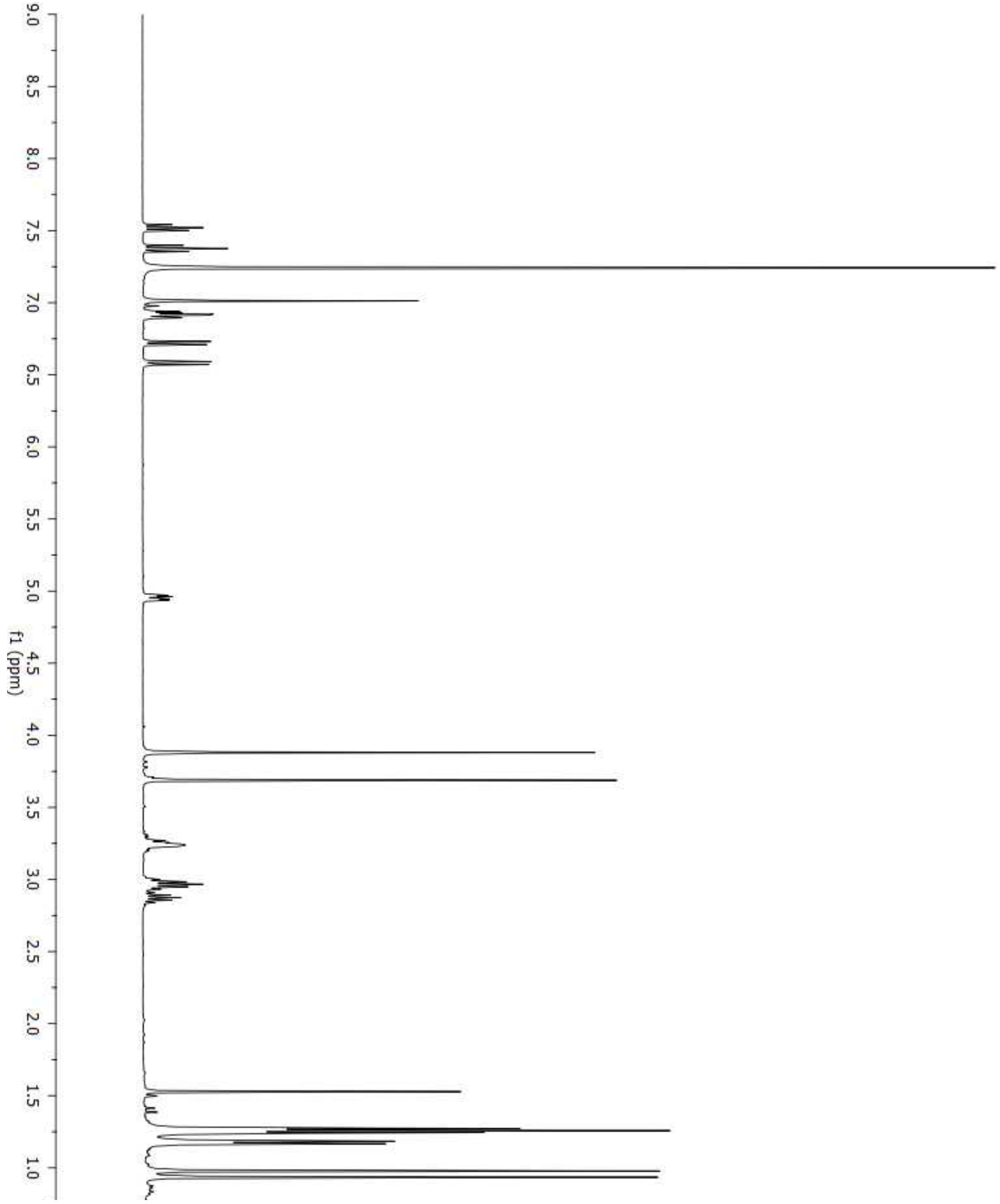

^{13}C NMR of (HandaPhos)AuCl

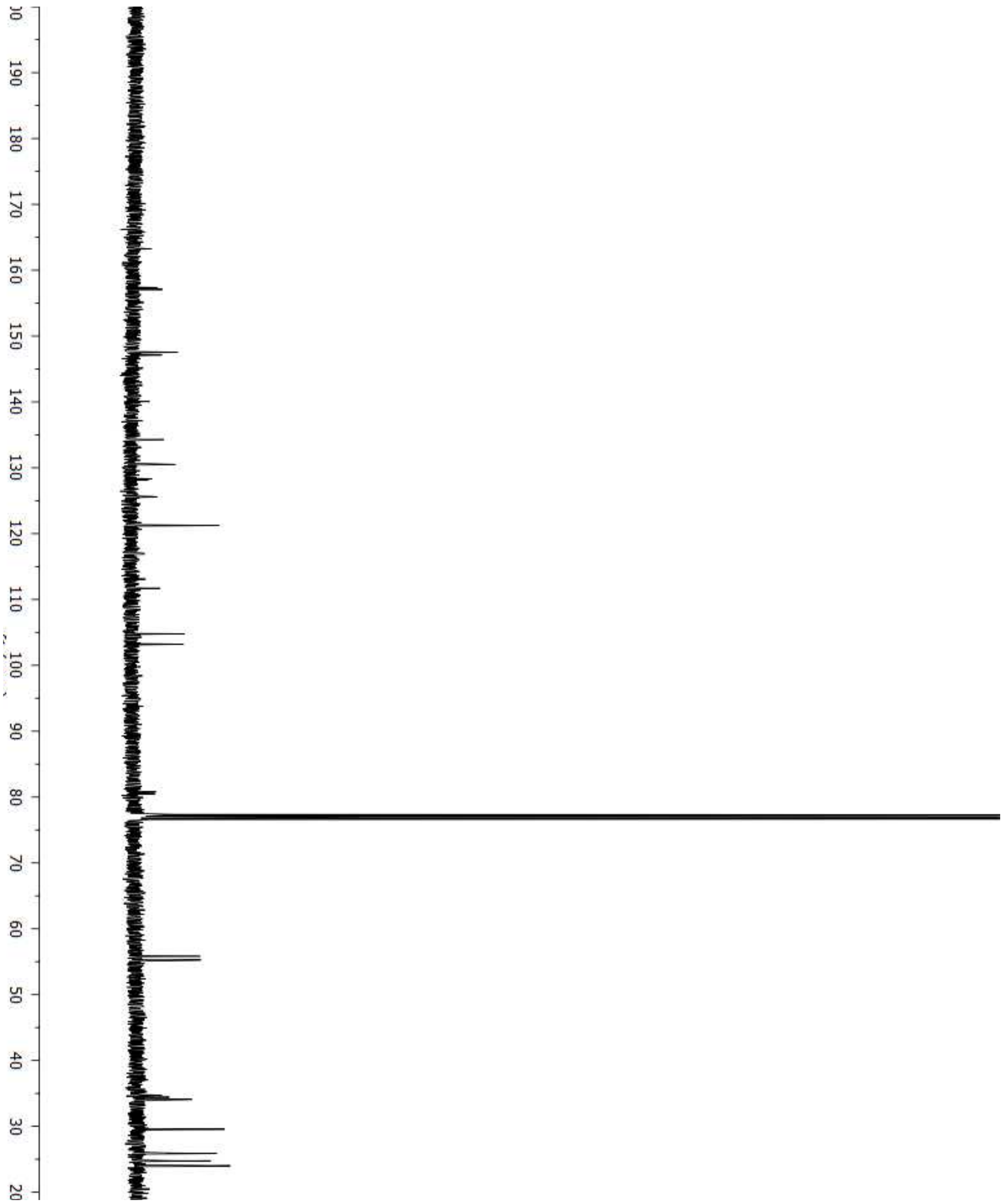

^{31}P NMR of (HandaPhos)AuCl

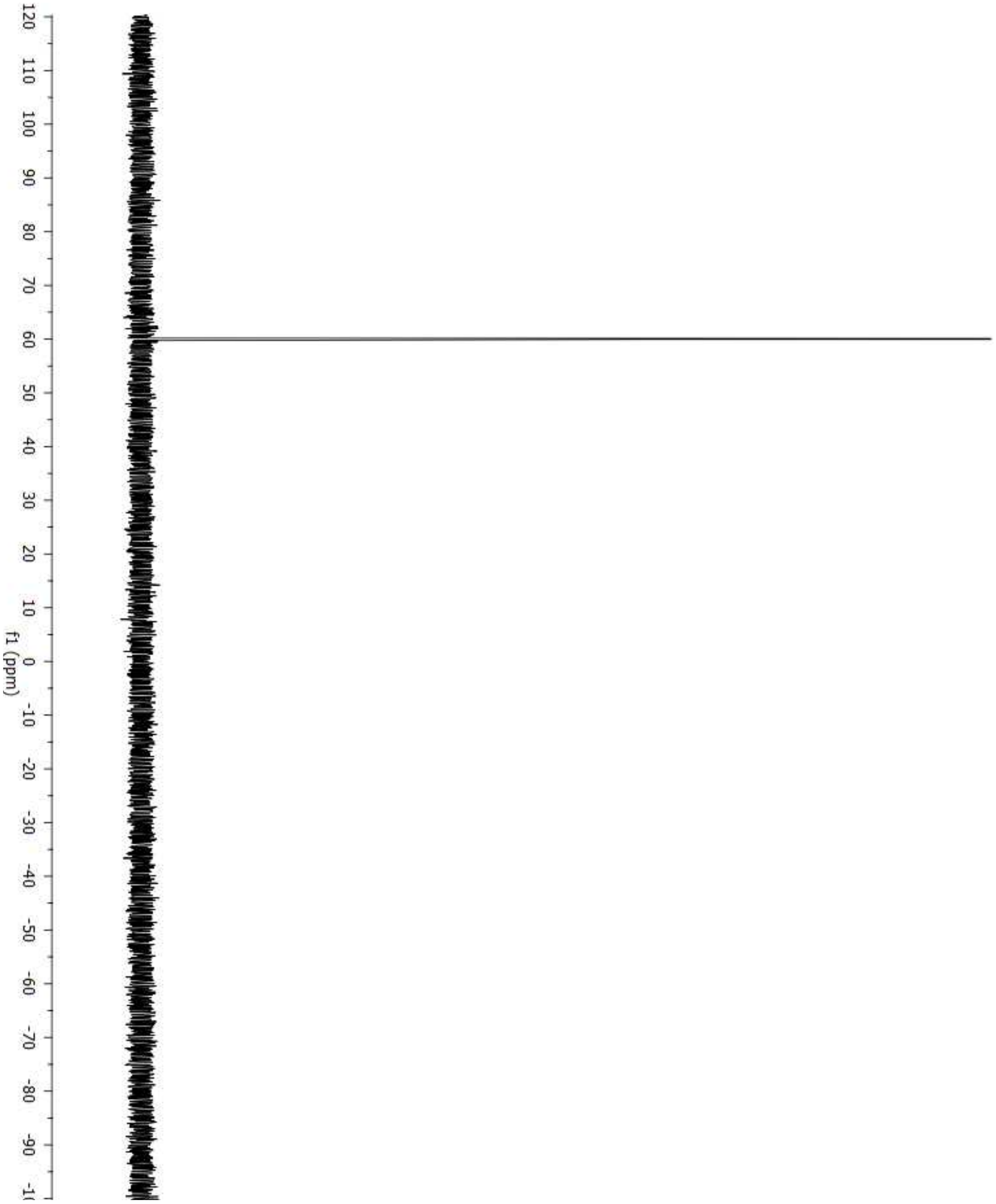

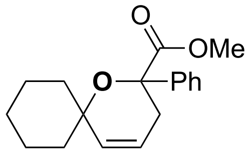

2

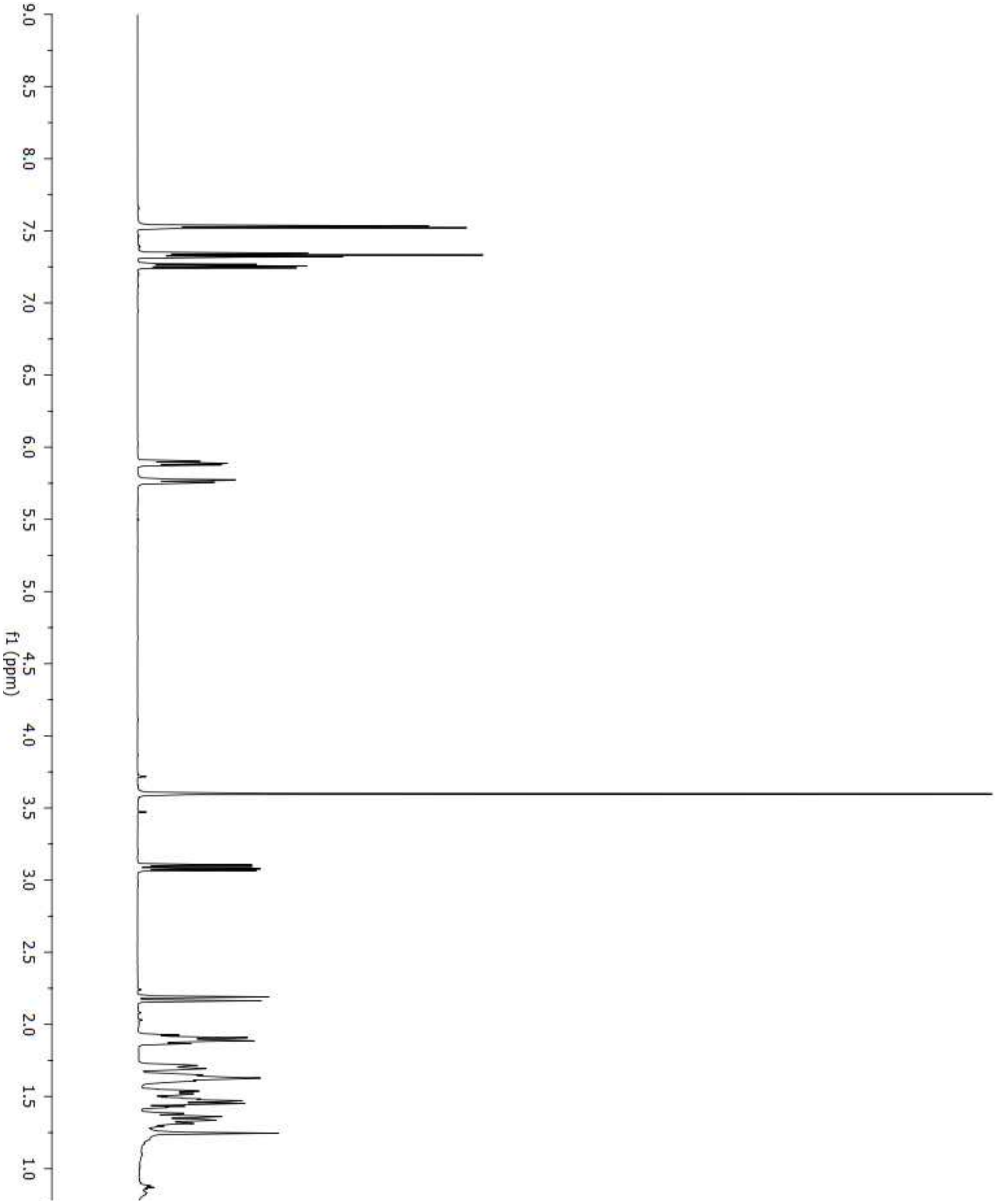

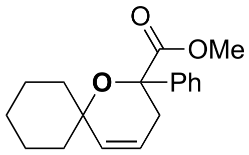
O
OMe
O
Ph

2

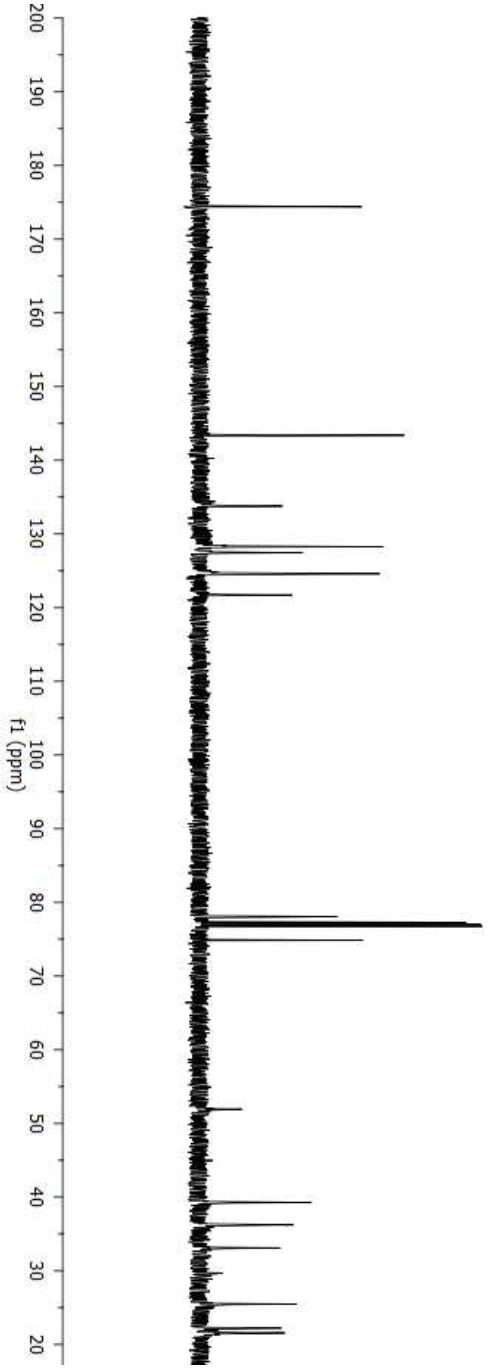
200
190
180
170
160
150
140
130
120
110
100
f1 (ppm)
90
80
70
60
50
40
30
20

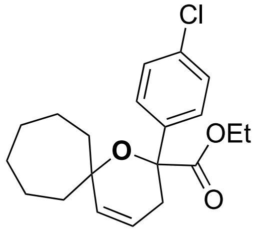

4

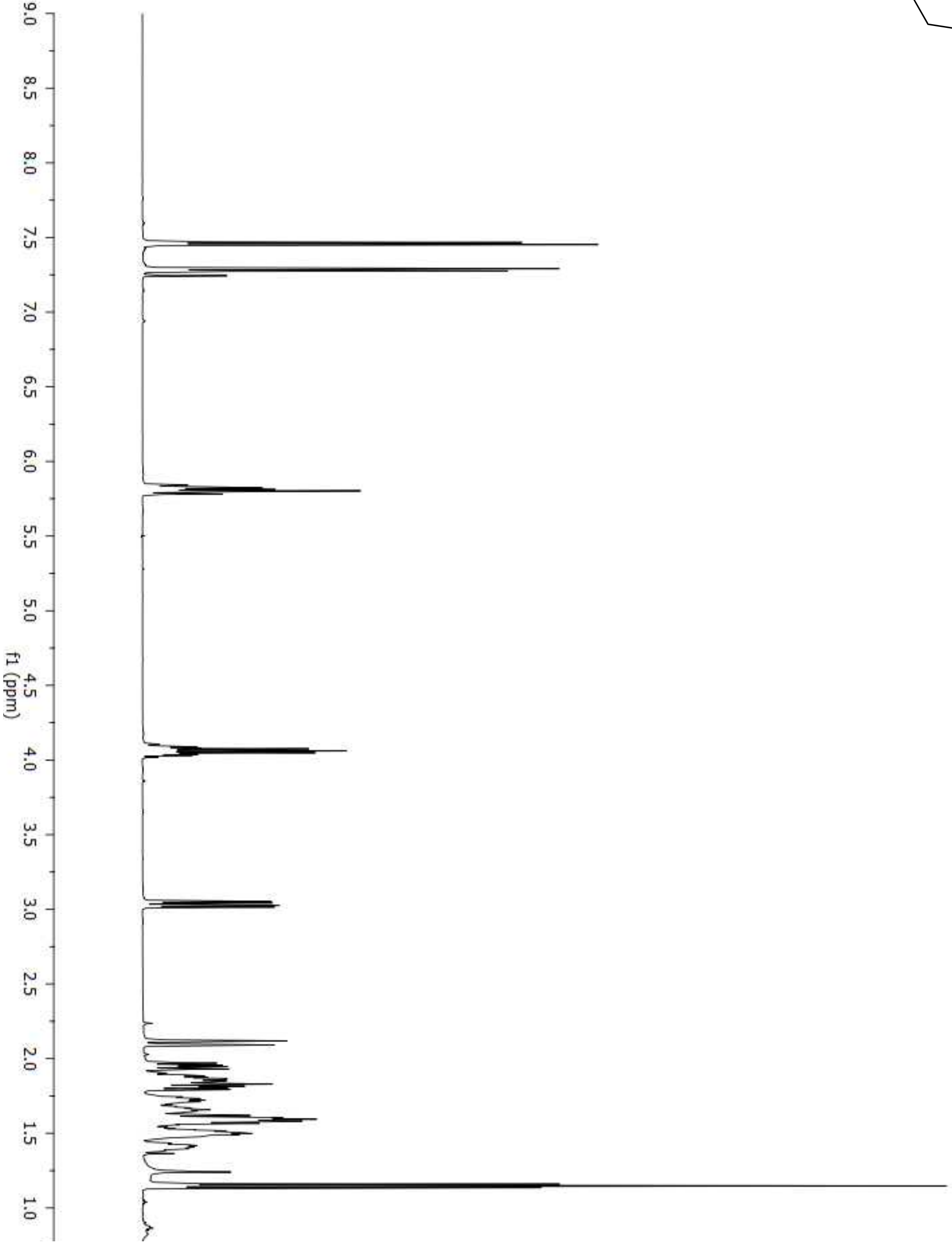

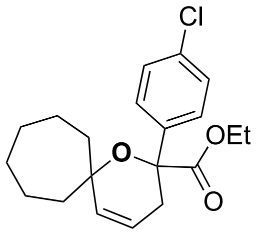
Cl
O
OEt
O

4

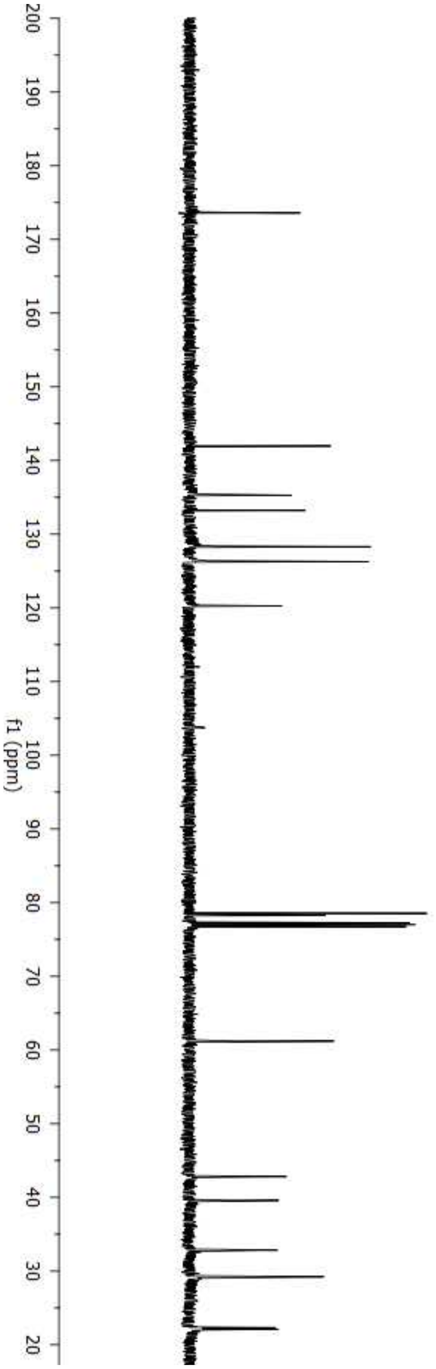
200
190
180
170
160
150
140
130
120
110
100
90
80
70
60
50
40
30
20
f1 (ppm)

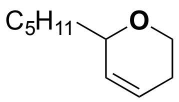

5

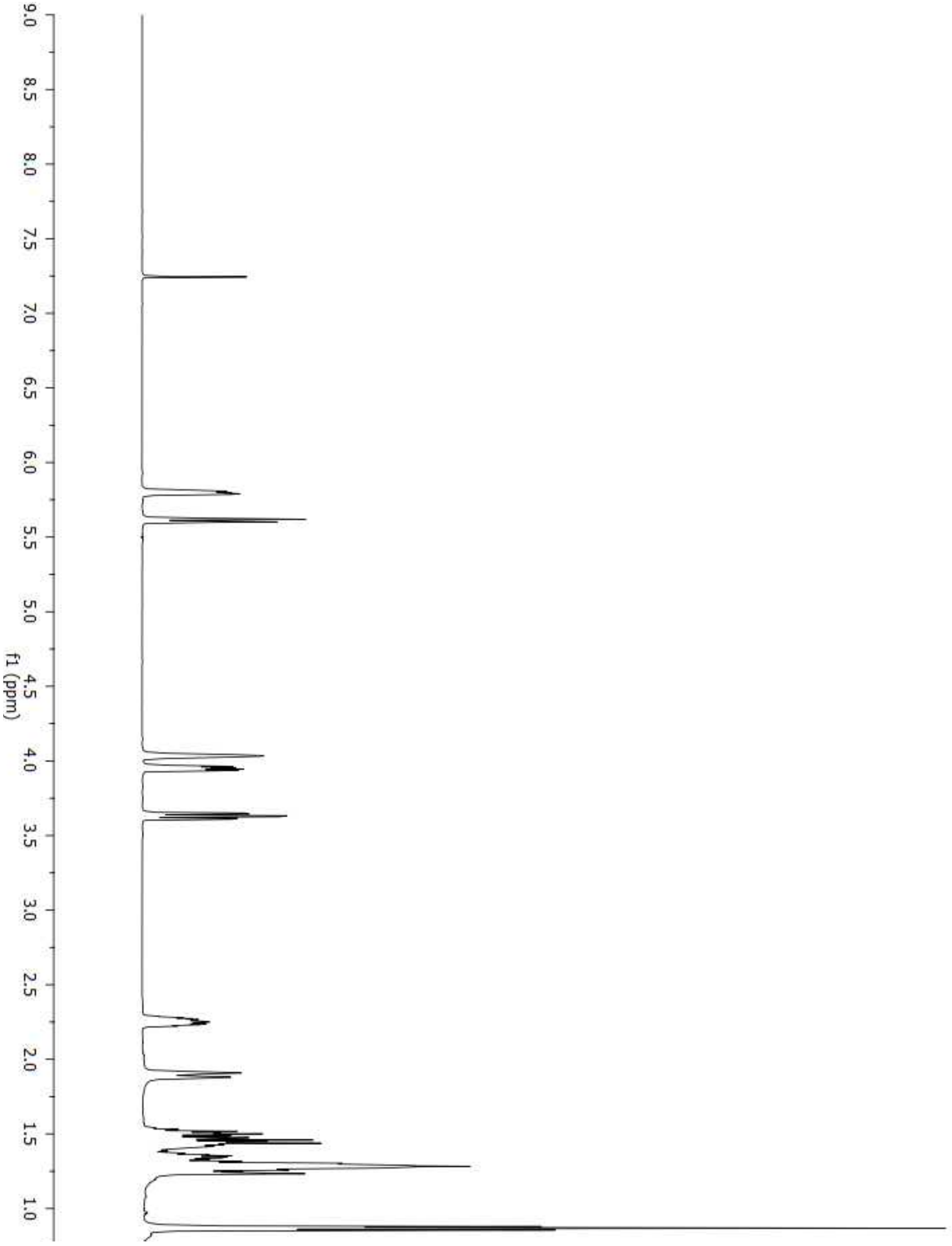

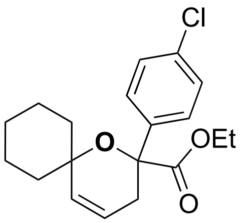

6

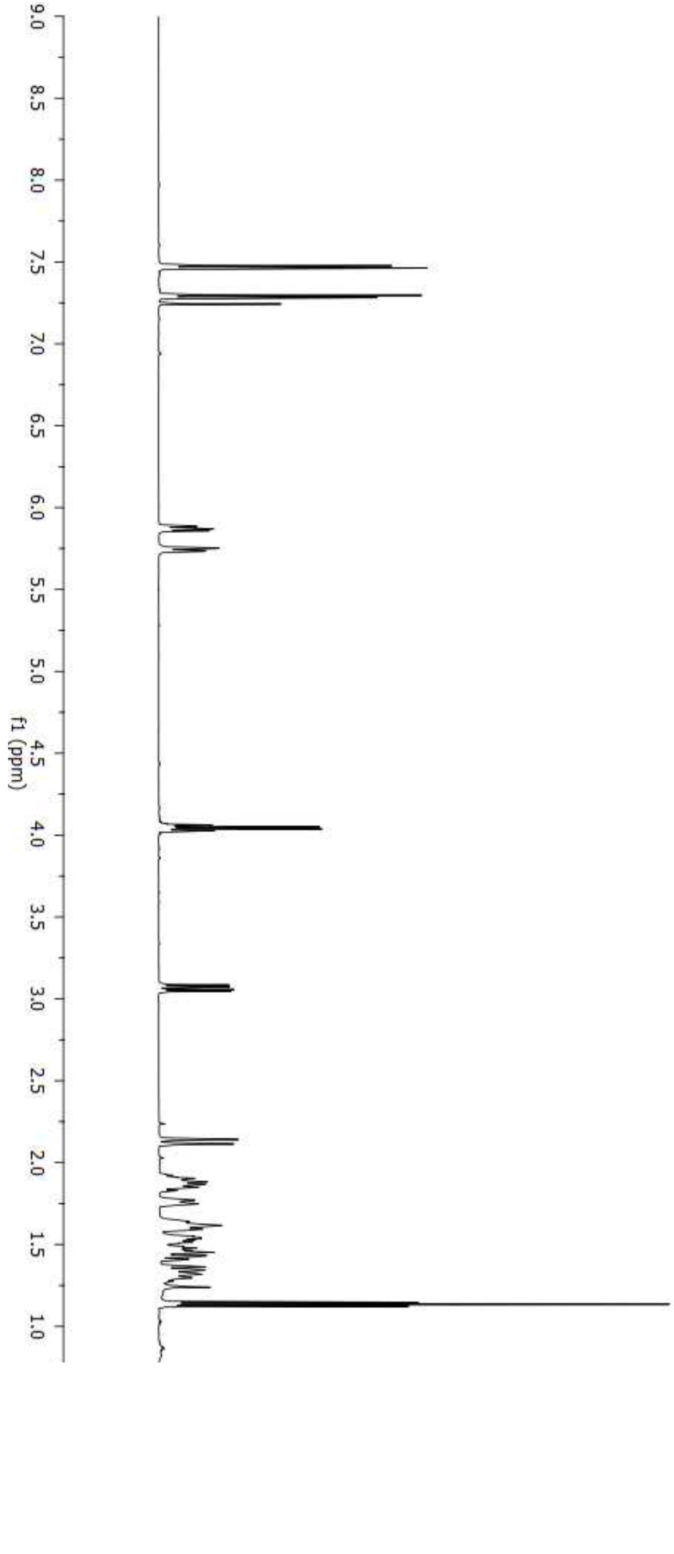

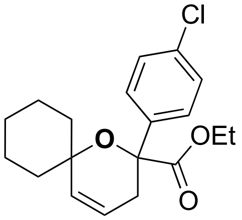
Cl
O
OEt
O

6

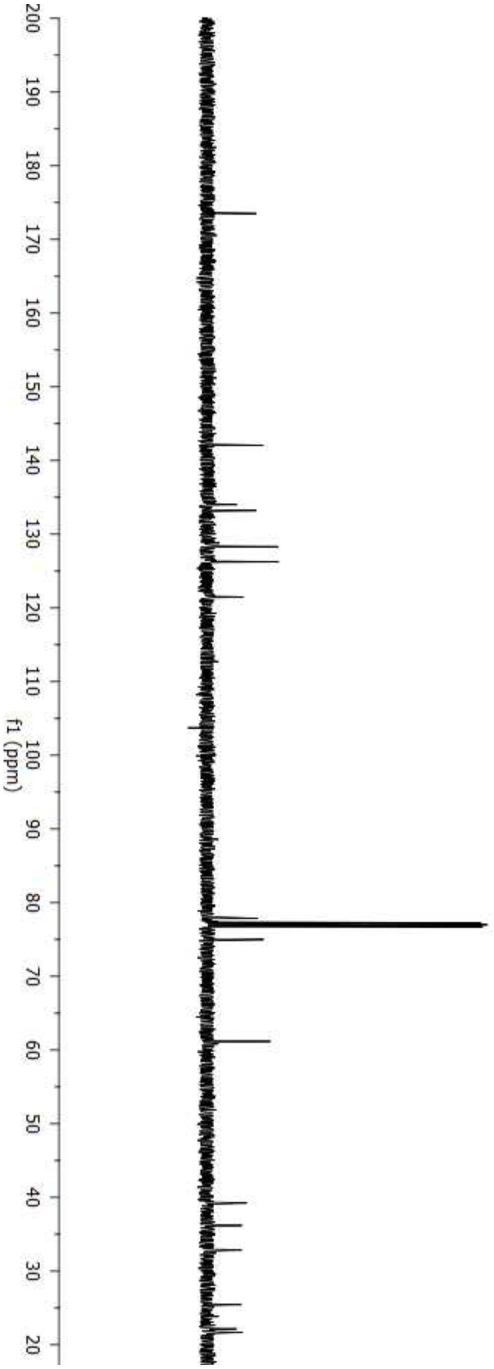
200
190
180
170
160
150
140
130
120
110
100
f1 (ppm)
90
80
70
60
50
40
30
20

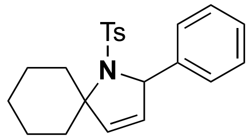

8

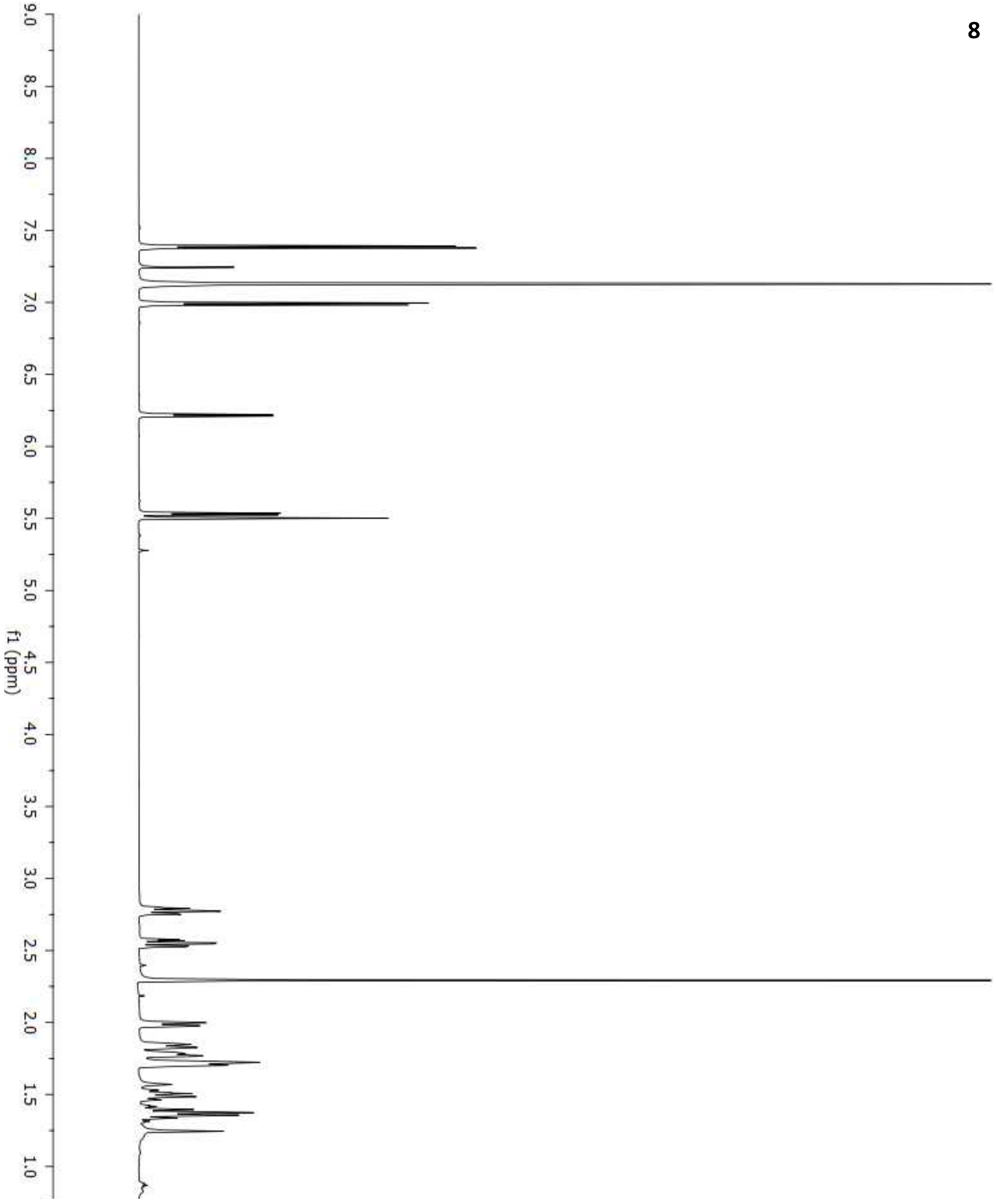

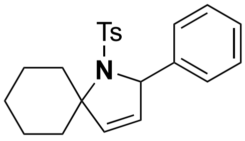

8

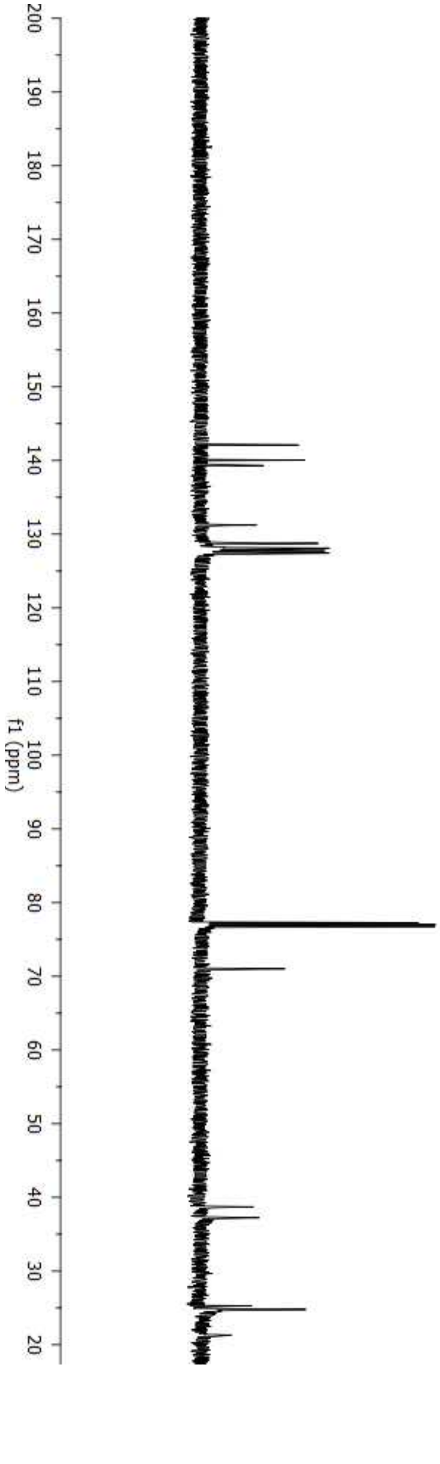

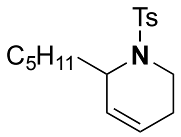

10

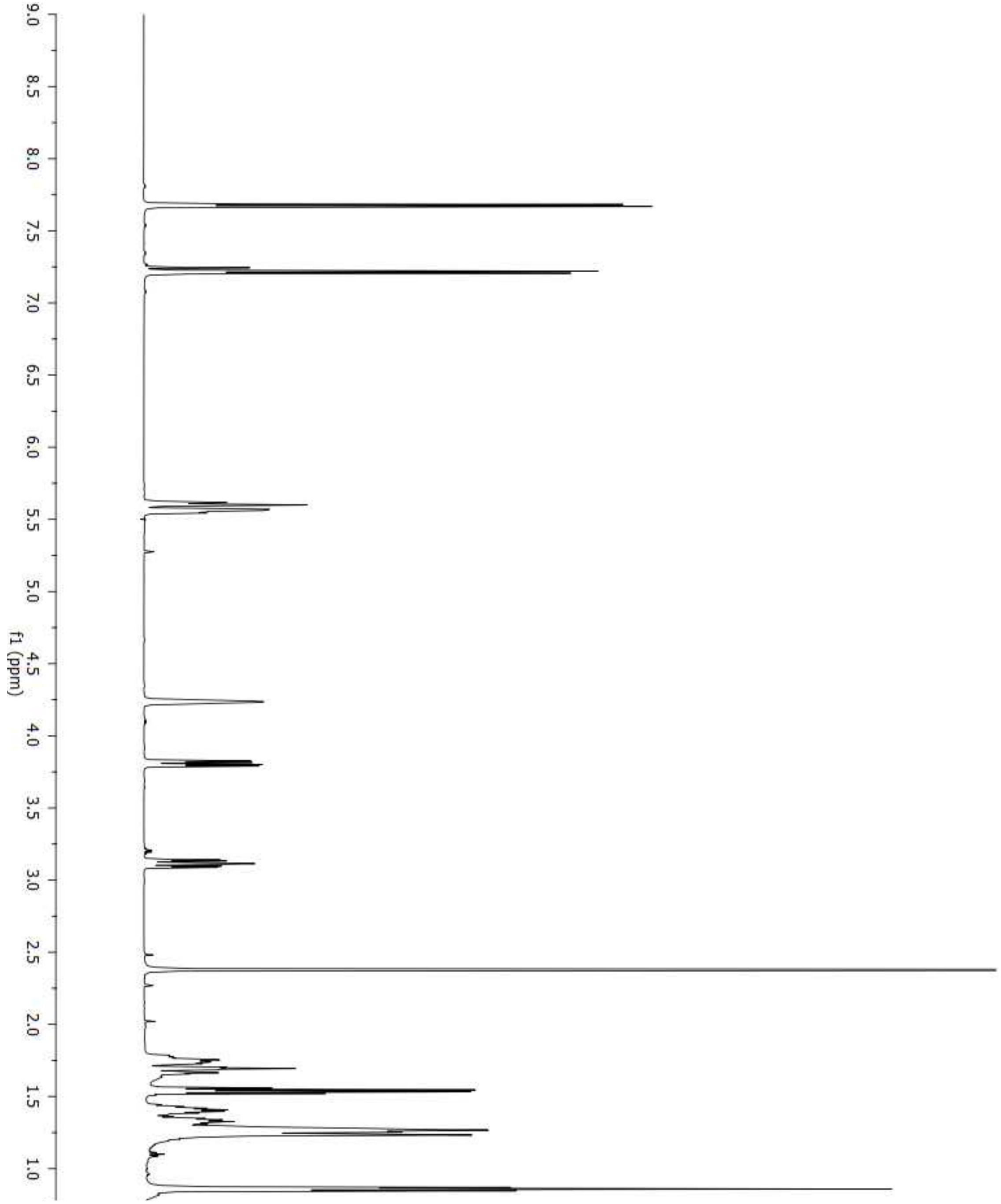

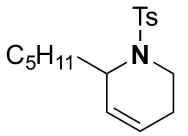
Ts
N
C_5H_{11}

10

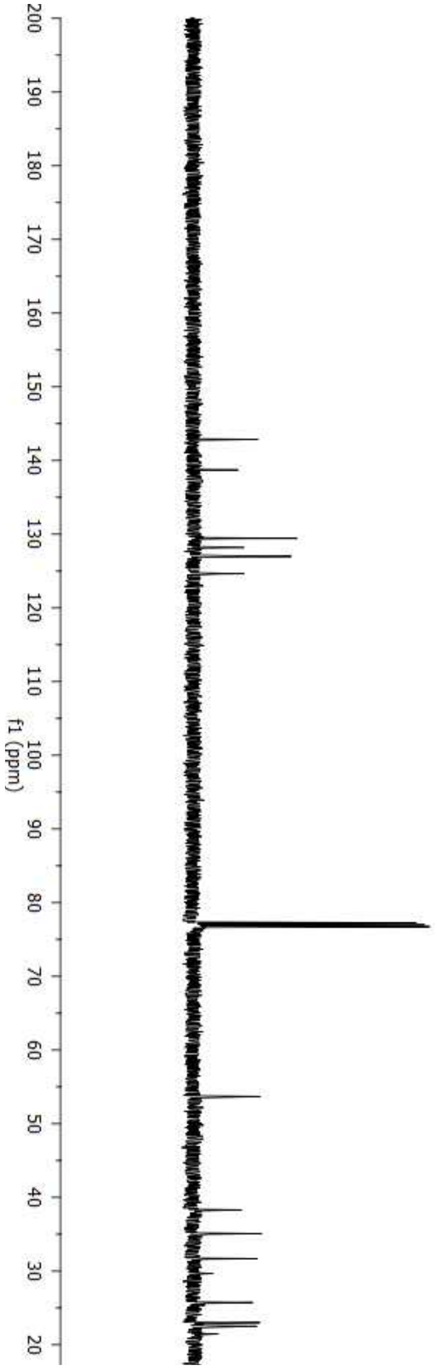
f1 (ppm)
200
190
180
170
160
150
140
130
120
110
100
90
80
70
60
50
40
30
20

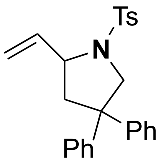

12

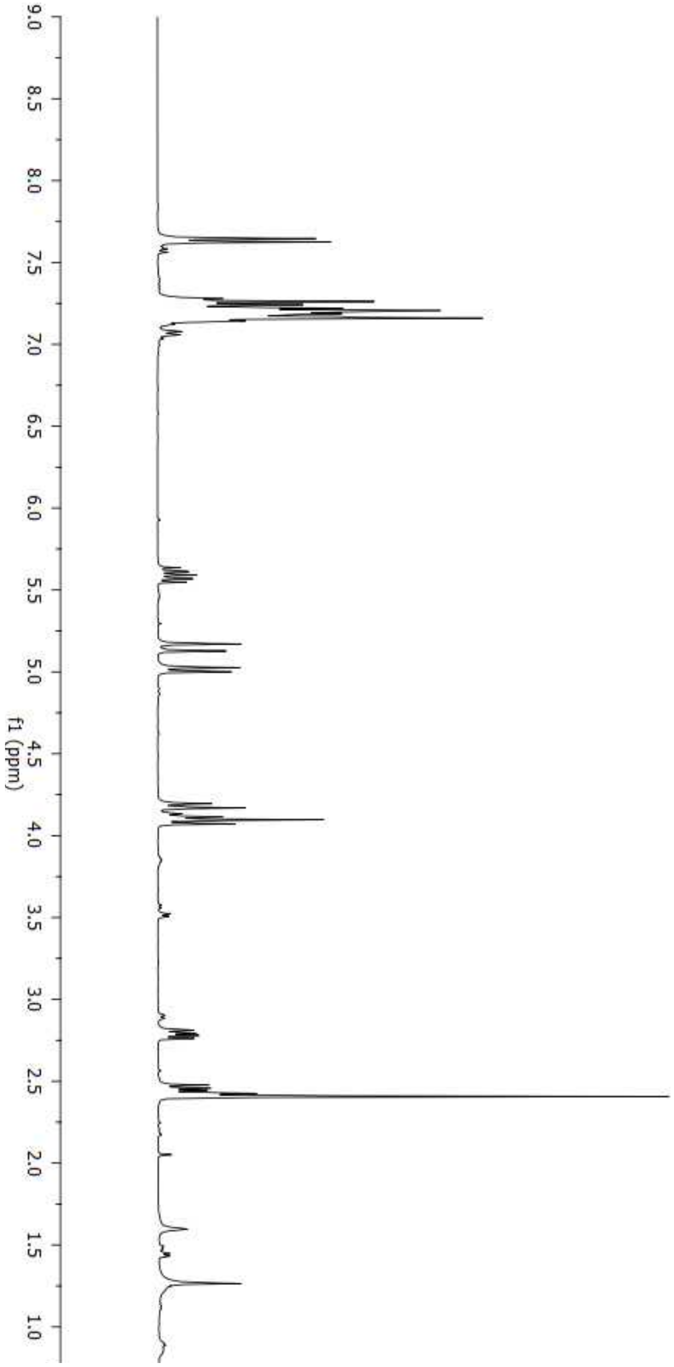

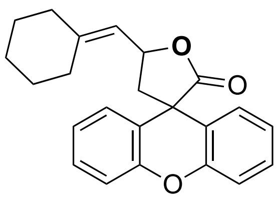

13

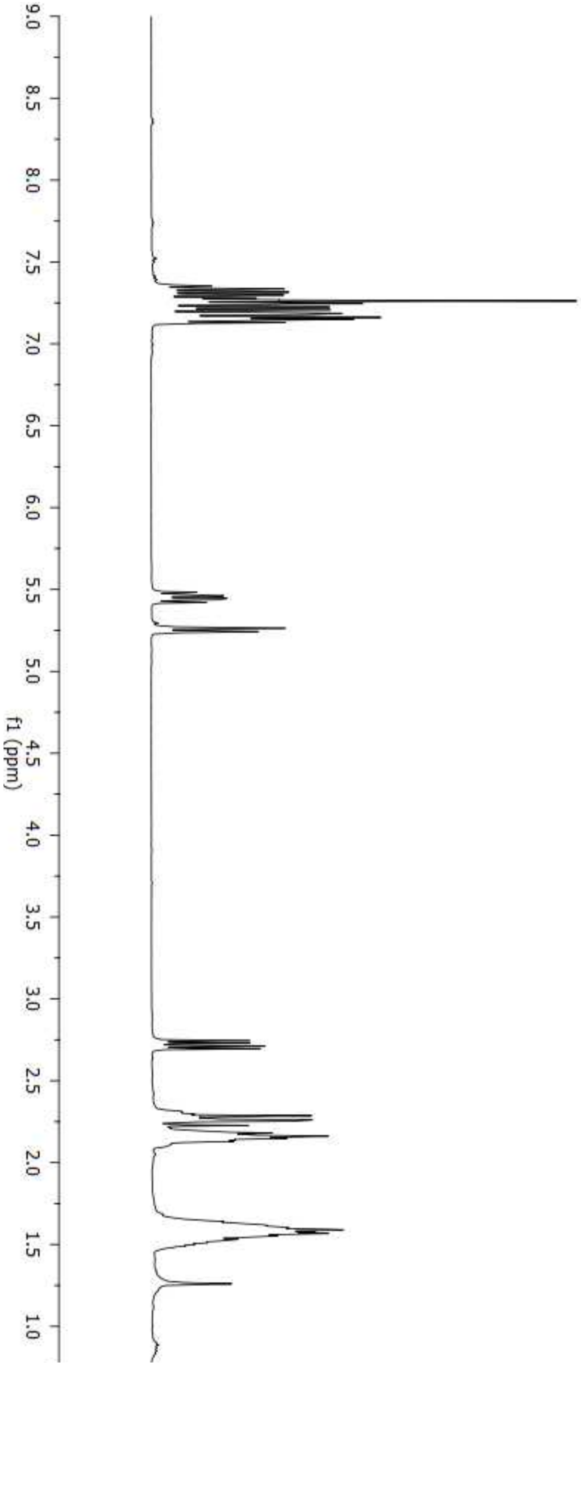

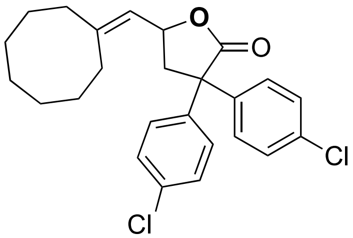

14

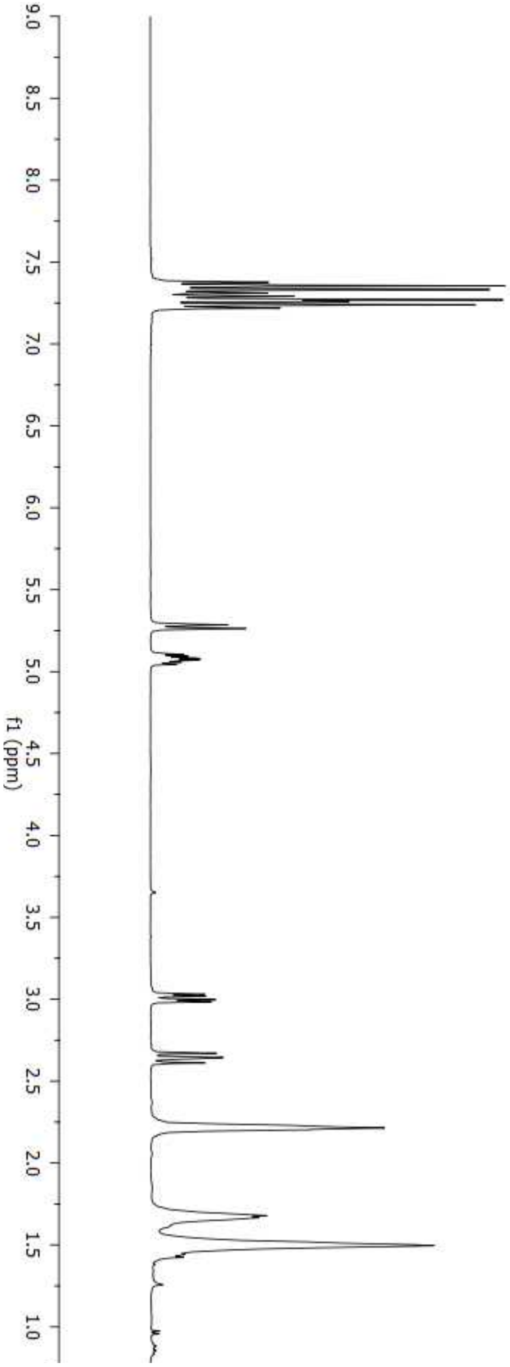

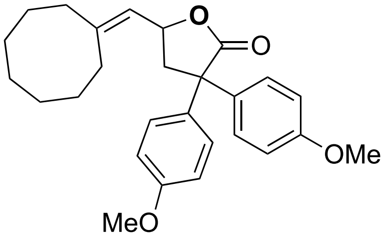

15

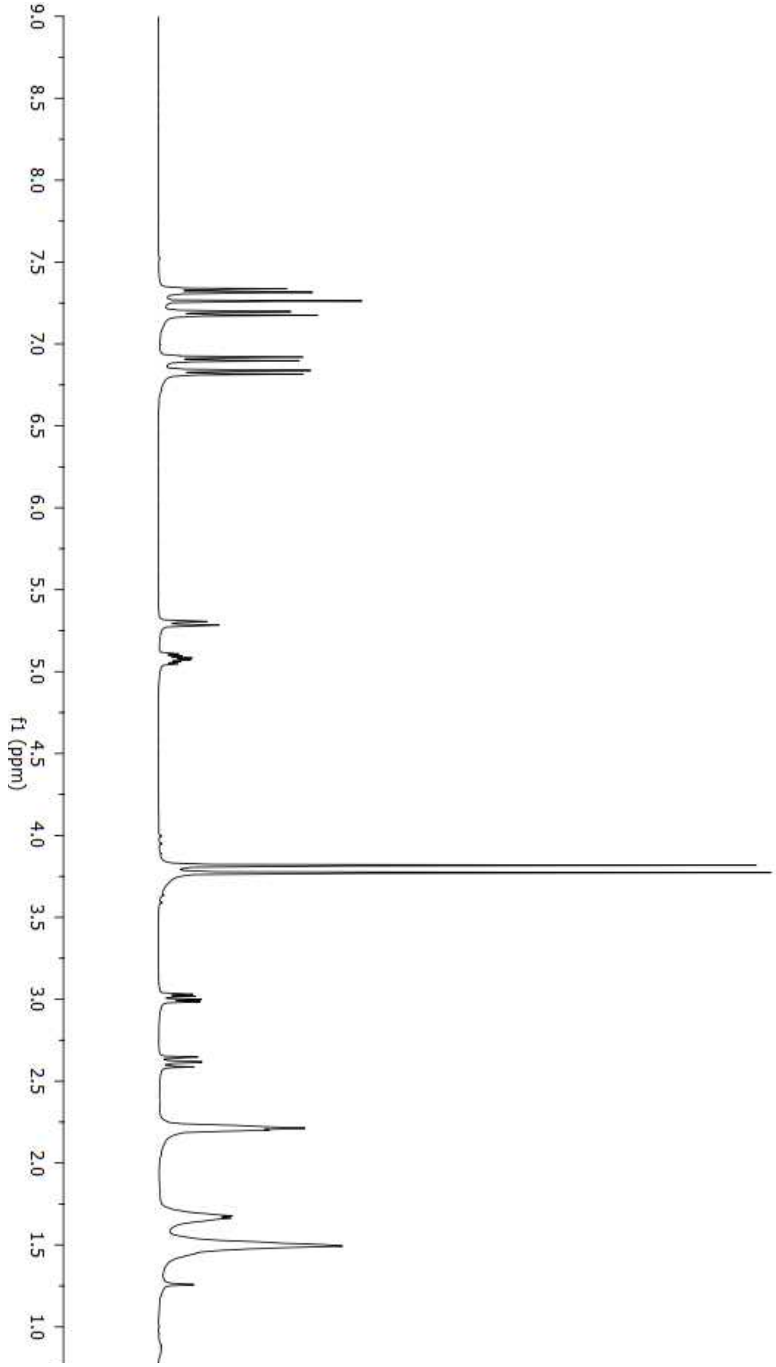

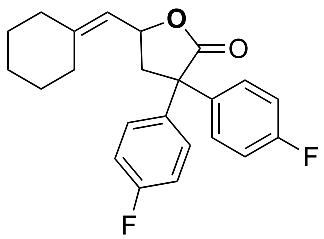

16

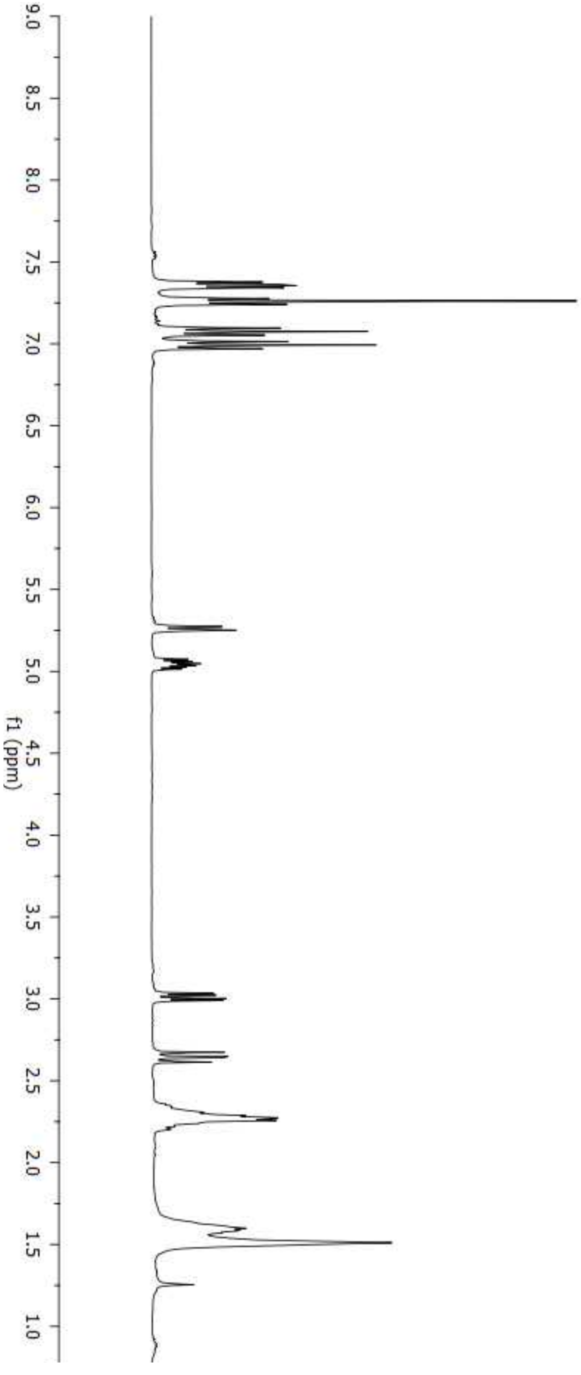

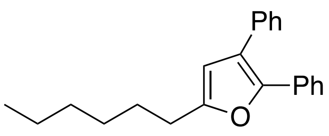

18

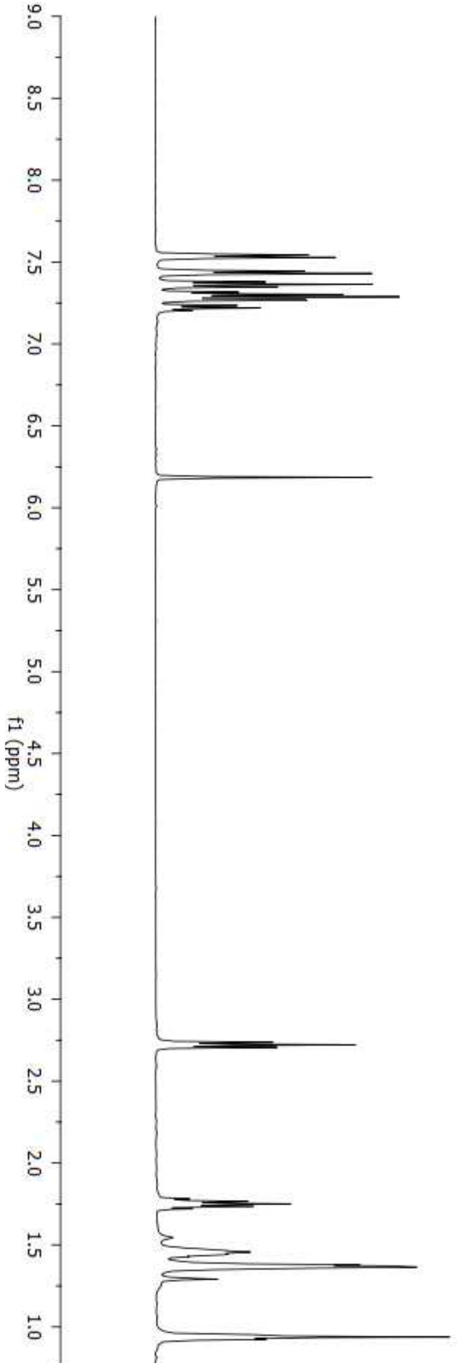

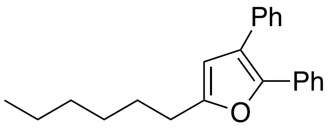
Ph
Ph
O

18

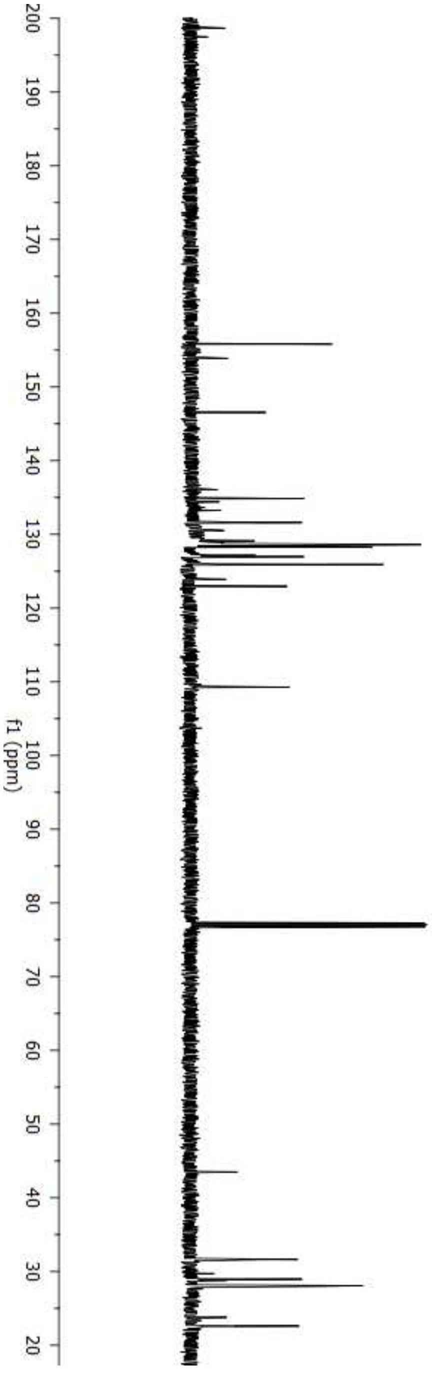
200
190
180
170
160
150
140
130
120
110
100
f1 (ppm)
90
80
70
60
50
40
30
20

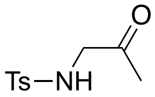

20

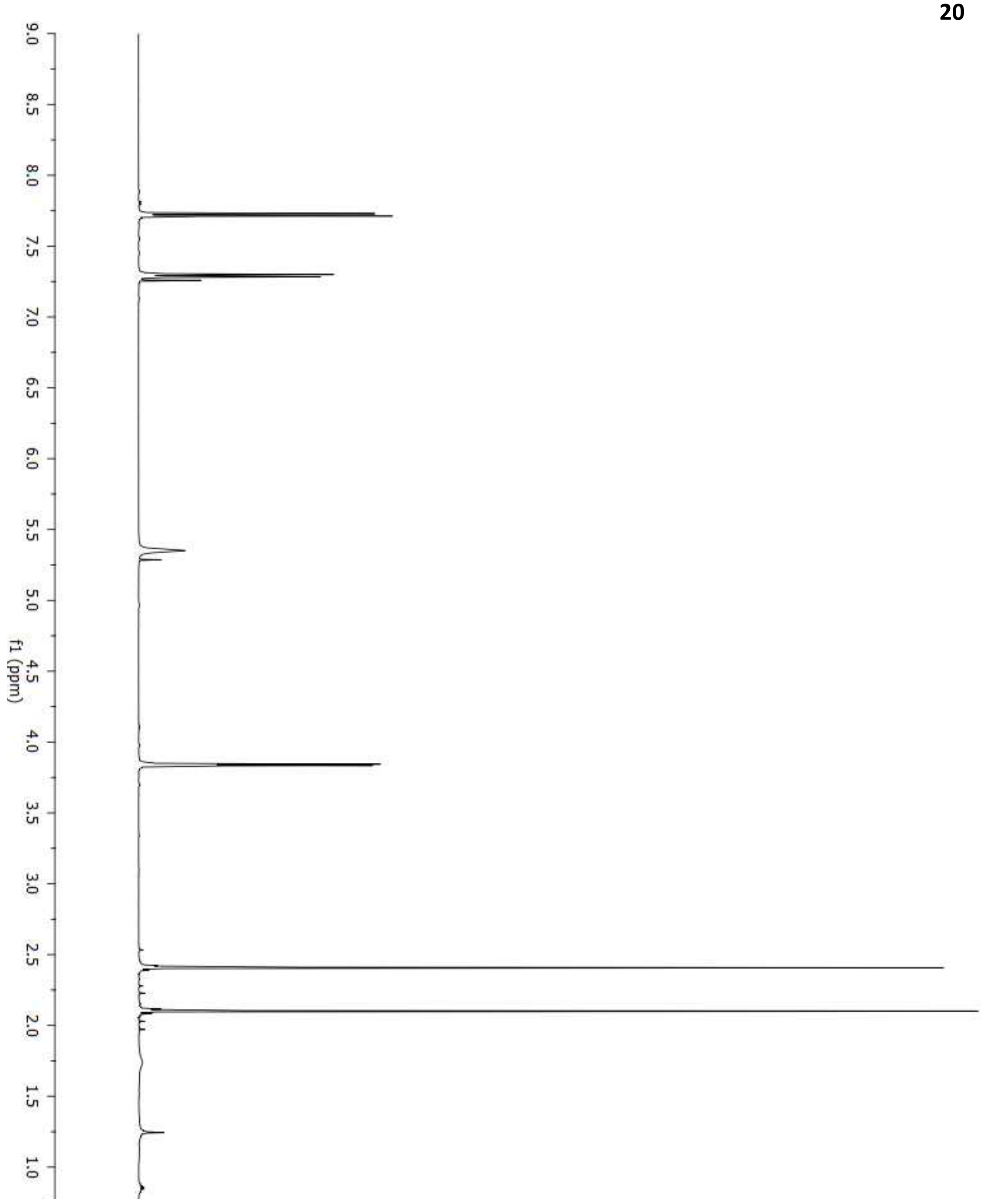

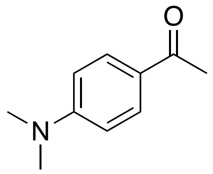

22

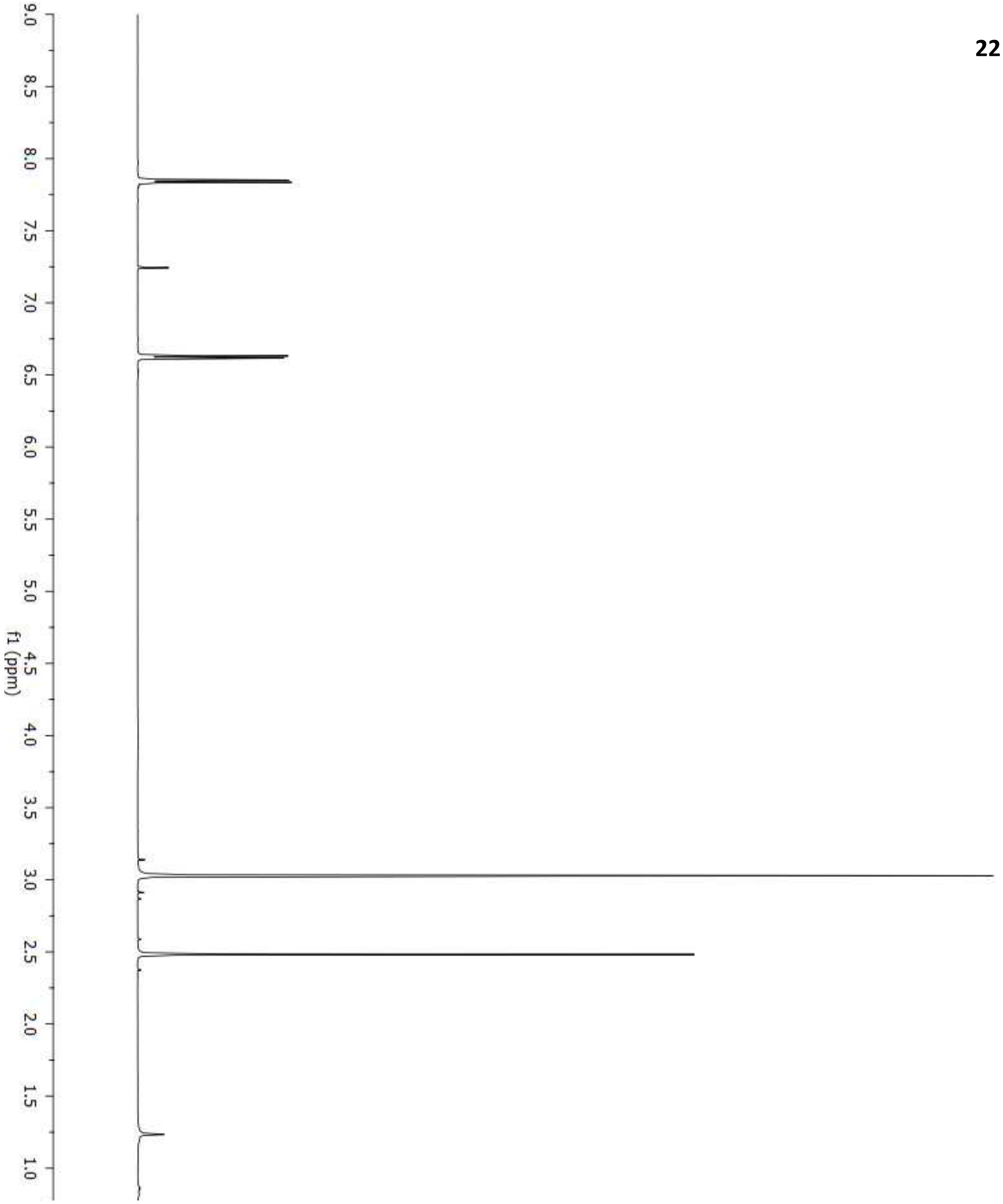

References

1. Handa, S.; Andersson, M. P.; Gallou, F.; Reilly, J.; Lipshutz, B. H. *Angew. Chem., Int. Ed.* **2016,** *55*, 4914.
2. Matousova, E.; Ruzicka, A.; Kunes, J.; Kralova, J.; Pour, M. *Chem. Commun.* **2011,** *47*, 9390.
3. Basu, S.; Waldmann, H. *J. Org. Chem.* **2006,** *71*, 3977.
4. Michon, C.; Medina, F.; Abadie, M.-A.; Agbossou-Niedercorn, F. *Organometallics* **2013,** *32*, 5589.
5. Handa, S.; Lippincott, D. J.; Aue, D. H.; Lipshutz, B. H. *Angew. Chem., Int. Ed.* **2014,** *53*, 10658.
6. Mizar, P.; Wirth, T. *Angew. Chem., Int. Ed.* **2014,** *53*, 5993.
7. Moskalev, N. V.; Zhuravkov, S. P.; Ogorodnikov, V. D. *Russ. Chem. Bull.* **1996,** *45*, 2461.
8. Soni, R.; Hall, T. H.; Mitchell, B. P.; Owen, M. R.; Wills, M. *J. Org. Chem.* **2015,** *80*, 6784.
